# Supplementary material for: Method to Synchronize Cell Cycle of Human Pluripotent Stem Cells without Affecting Their Fundamental Characteristics
Source: Stem Cell Reports. 2018 Dec 27;12(1):165–79. doi: 10.1016/j.stemcr.2018.11.020 (PMC6335580; doi:10.1016/j.stemcr.2018.11.020)
Supplement: Document S1. Supplemental Experimental Procedures and Figures S1–S4 [file mmc1.pdf]

Stem Cell Reports, Volume *12*

Supplemental Information

Method to Synchronize Cell Cycle of Human Pluripotent Stem Cells without Affecting Their Fundamental Characteristics

Loukia Yiangou, Rodrigo A. Grandy, Carola M. Morell, Rute A. Tomaz, Anna Osnato, Juned Kadiwala, Daniele Muraro, Jose Garcia-Bernardo, Shota Nakanoh, William G. Bernard, Daniel Ortmann, Davis J. McCarthy, Ingrid Simonic, Sanjay Sinha, and Ludovic Vallier

SUPPLEMENTAL INFORMATION

Supplemental Figures

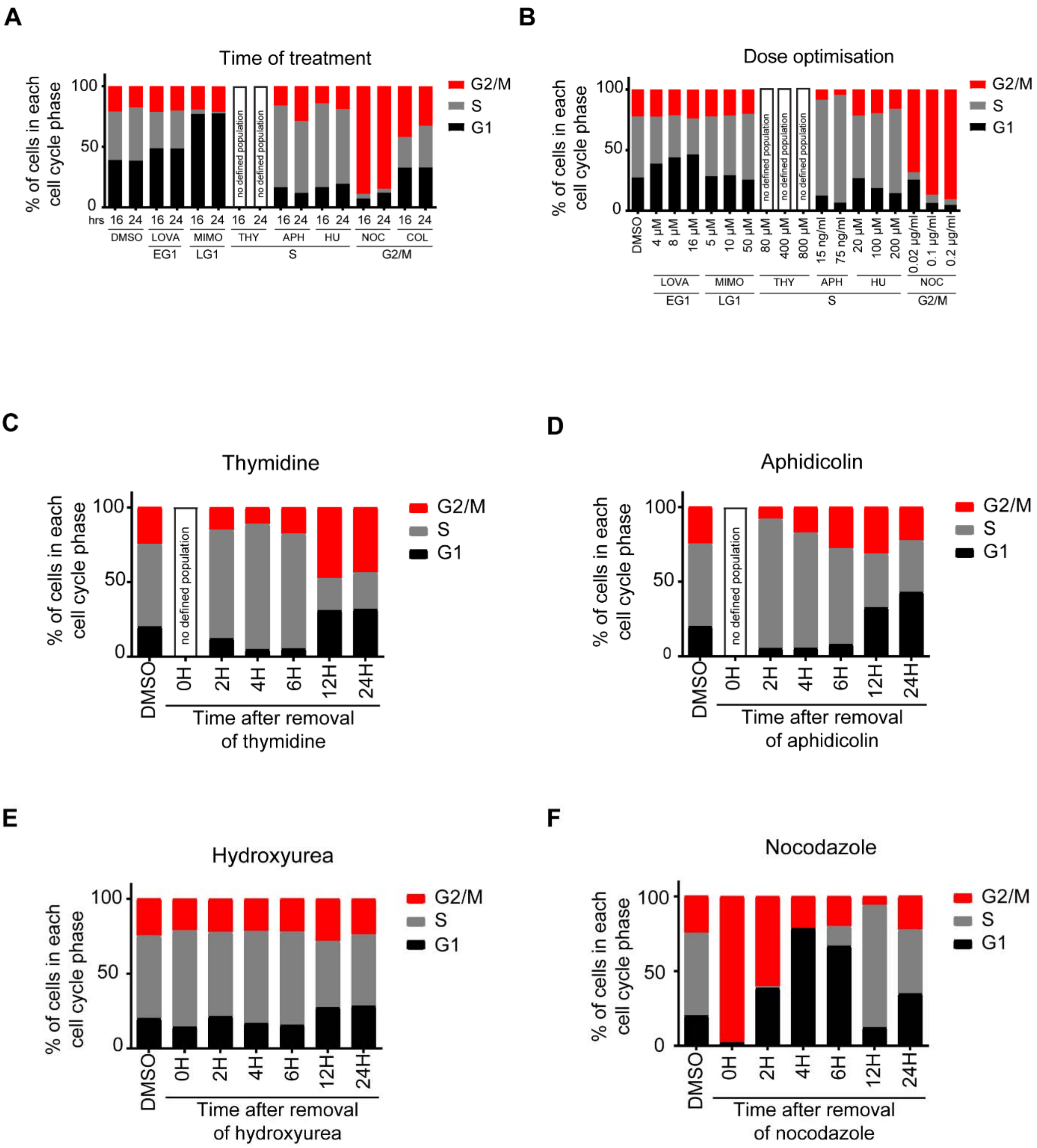

Figure S1. Optimisation of timing and dose of small molecule cell cycle inhibitor treatment. Related to Figure 1.

(A) Cell cycle profile of H9 hESCs, incubated for 16 or 24 hours with the small molecule cell cycle inhibitors.

(B) Cell cycle profile of H9 hESCs, incubated with different doses of the small molecule cell cycle inhibitors.

(C-F) Cell cycle profile of H9 hESCs following treatment and removal of cell cycle inhibitors thymidine (C), aphidicolin (D), hydroxyurea (E) and nocodazole (F) through a timecourse of 24 hours.

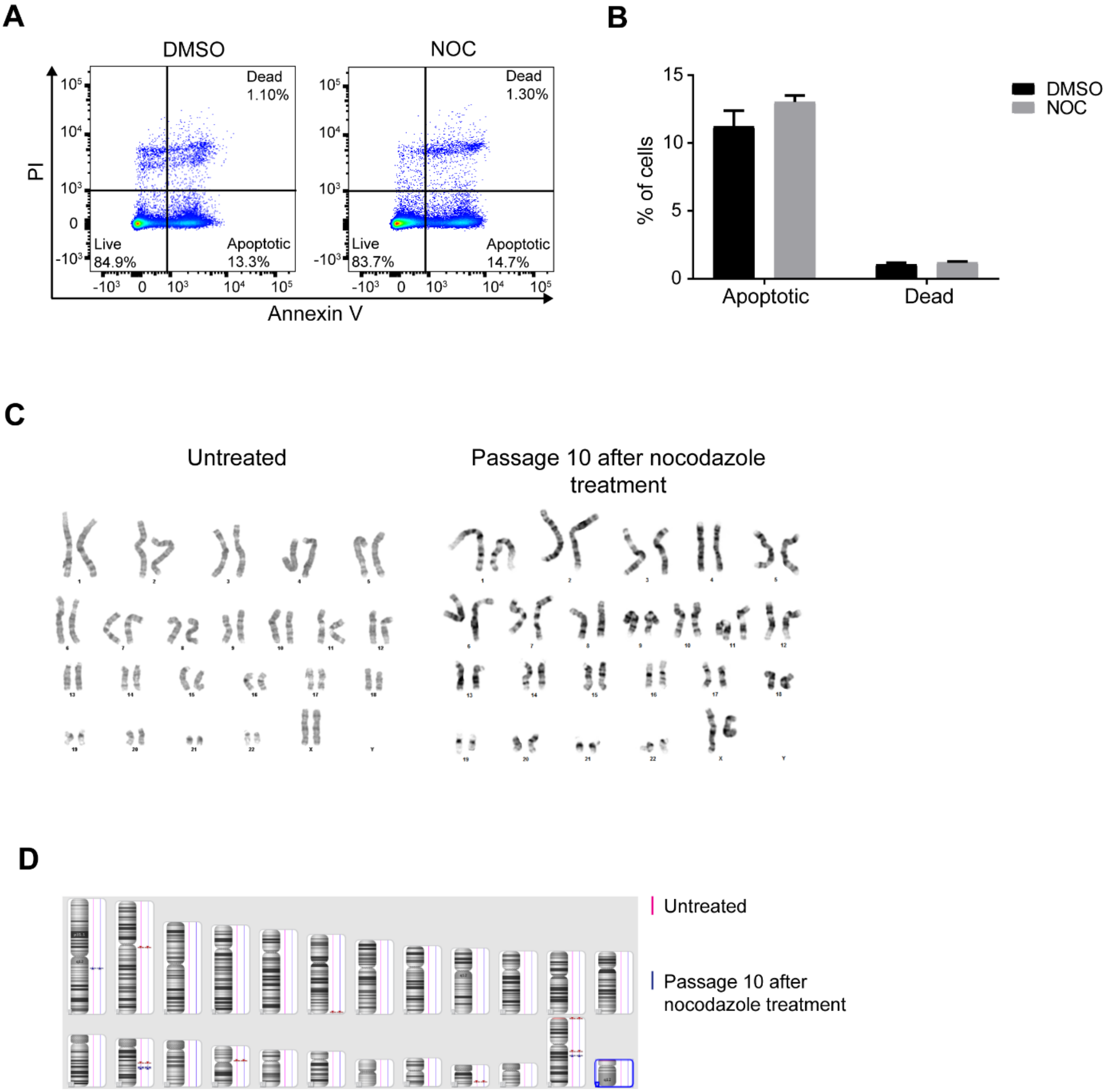

Figure S2. Nocodazole treatment does not cause karyotypic abnormalities in hESCs. Related to Figure 3.
(A) Representative flow cytometry analysis for Annexin V and Propidium iodide (PI) positive cells. Annexin V+/PI- cells are apoptotic and Annexin V+/PI+ cells are dead.
(B) Bar graph summarising flow cytometry results of Annexin V/PI staining. Error bars represent ±SEM of two independent experiments.
(C) Chromosomal spreads showing normal karyotype in untreated and nocodazole-treated H9 hESCs after ten passages in culture.
(D) Karyoview representation of CytoScan 750K array analysis comparing untreated (pink) and nocodazole treated (blue) H9 hESCs after ten passages in culture.

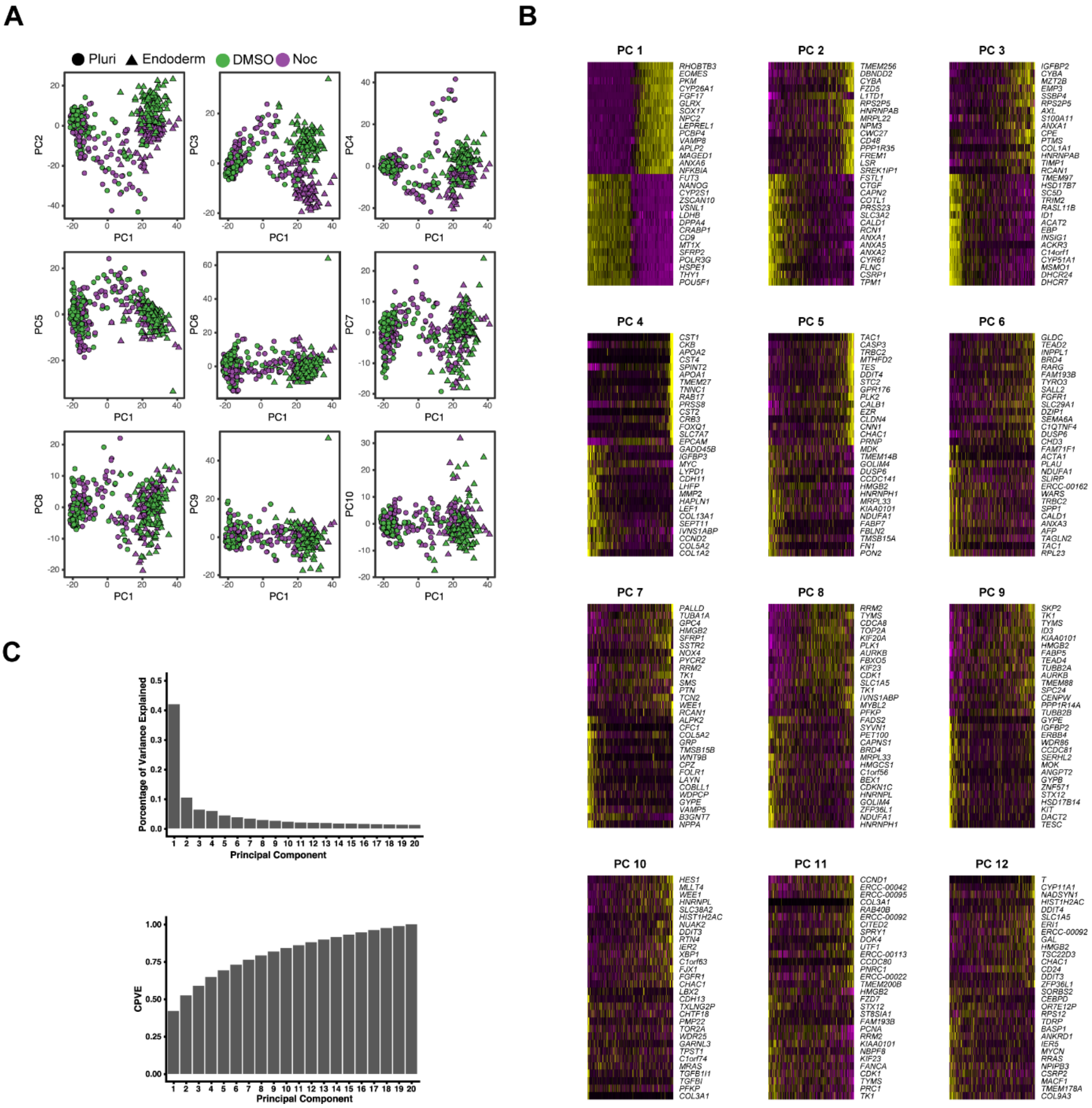

Figure S3. Single Cell RNA-Seq Confirms That Nocodazole Treatment Does Not Affect the Ability of Pluripotent Cells to Differentiate into Definitive Endoderm. Related to Figure 4.

(A) Principal component analysis (PCA) plot showing the assignment of cells based on differentiation and synchronization status. Normalized log-expression values were used. Dots represent individual cells. (DMSO = Green, Noc = Purple, Und = Circle, Endoderm = Triangle).

(B) Heatmaps showing the top 500 cells and 30 genes sorted by their first 12 principal component scores. These plots allow for visualisation of sources of heterogeneity in the dataset.

(C) Graphs showing the percentage of variance explained and the cumulative proportion of variance explained (CPVE).

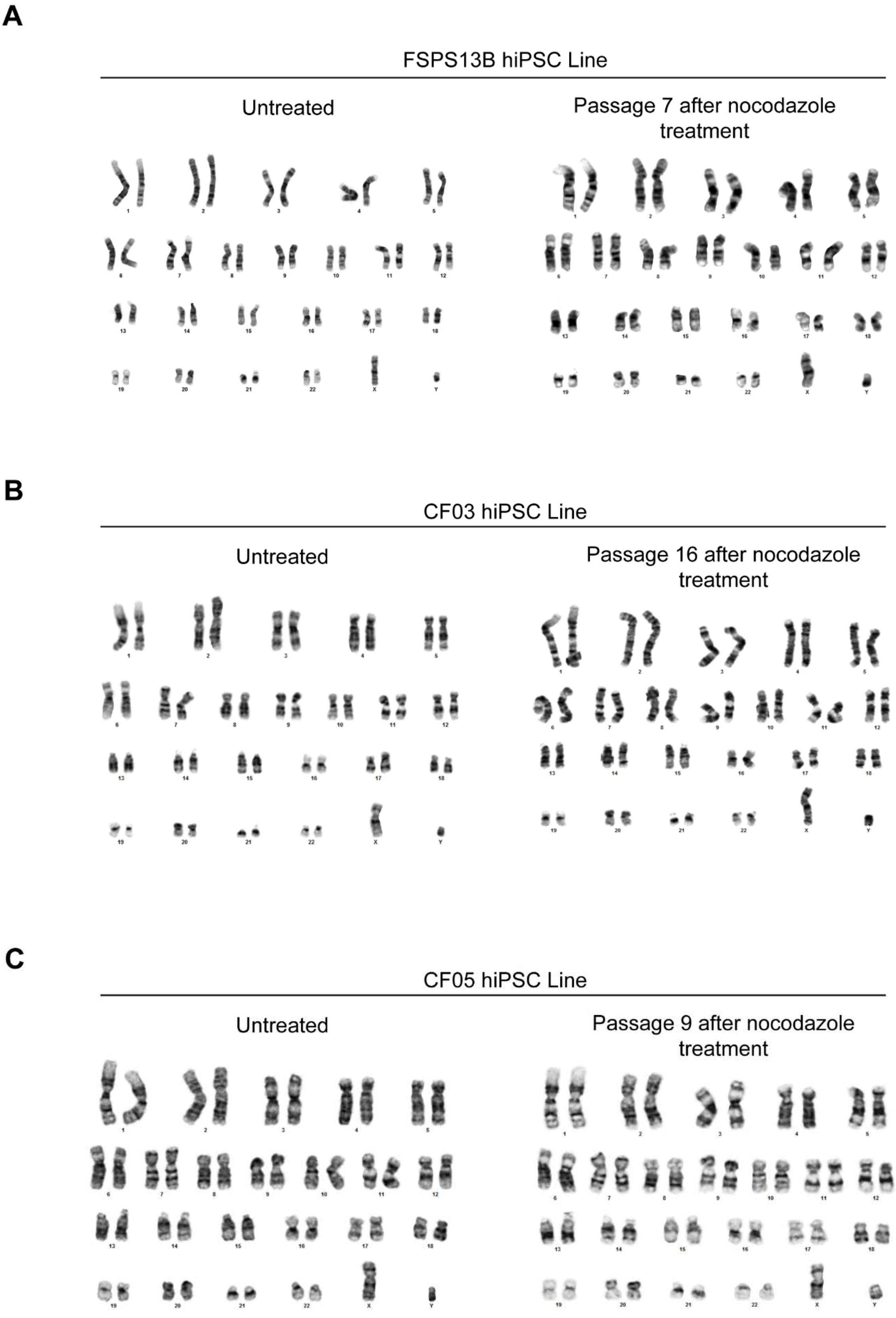

Figure S4. Nocodazole treatment does not cause karyotypic abnormalities in hiPSCs. Related to Figure 7. (A-C) Chromosomal spreads showing normal karyotype for both untreated and nocodazole treated FSPS13B line (A), CF03 line (B), and CF05 line (C).

Supplemental Experimental Procedures

Germ layer Differentiation

Mesoderm subtypes were generated in a 2-step protocol as previously described (Mendjan et al., 2014). For lateral plate mesoderm (LPM) formation, cells were cultured for 36 hours in CDM-PVA supplemented with 20ng/ml FGF2, 10µM LY294002 (Promega) and 10ng/ml BMP4 (R&D). Subsequently cells were cultured for 3 days in CDM-PVA supplemented with 20ng/ml FGF2 and 50ng/ml BMP4 changing medium every two days. For cardiac mesoderm (CM) formation, cells were cultured for 36 hours in CDM-BSA (without insulin) supplemented with 20ng/ml FGF2, 10µM LY294002, 10ng/ml BMP4 and 50ng/ml Activin A (Dr. Marko Hyvönen, Cambridge University). Subsequently cells were cultured for 4 days in CDM-BSA (without insulin) supplemented with 8ng/ml FGF2, 10ng/ml BMP4, 1µM IWR1 (WNT signalling inhibitor; Tocris Bioscience) and 0.5µM Retinoic Acid (Sigma-Aldrich), changing medium every two days. For presomitic mesoderm (PSM) formation, cells were cultured for 36 hours in CDM-BSA (without insulin) supplemented with 20ng/ml FGF2 and 8µM CHIR99021 (WNT signalling activator; Tocris Bioscience). Subsequently cells were cultured for 4 days in CDM-BSA (with insulin) supplemented with 4ng/ml FGF2, 1µM Retinoic Acid, 0.1µM LDN193189 (BMP signalling inhibitor; Sigma-Aldrich) and 10µM SB431542 (TGF-β signalling inhibitor; Tocris Bioscience). For mesoderm differentiation cells were plated on gelatin and mouse embryonic fibroblast (MEF) medium coated plates.

Definitive endoderm was generated using a 3-day protocol. On day 1 cells were cultured in CDM-PVA supplemented with 80ng/ml FGF2, 10µM LY294002, 10ng/ml BMP4, 100ng/ml Activin A and 3µM CHIR99021. On day 2 the cells were cultured in CDM-PVA supplemented with 80ng/ml FGF2, 10µM LY294002, 10ng/ml BMP4 and 100ng/ml Activin A. On day 3 the cells were cultured in RPMI-B27 media supplemented with 80ng/ml FGF2 and 100ng/ml Activin. For endoderm differentiation cells were plated on vitronectin coated plates (10µg/ml, Stem Cell Technologies).

Neuroectoderm was generated using a 7-day protocol. On days 1 and 2 the cells were cultured in CDM-BSA supplemented with 20ng/ml FGF2, 3µM CHIR99021, 0.1µM LDN193189 and 10µM SB431542, changing medium daily. From day 3 to day 6 cells were cultured in Neurobasal medium/DMEM F12 (1:1 ratio) supplemented with N2-B27, 1% glutamine, 10µM SB431542 and 0.7% β-mercaptoethanol, changing medium daily.

Smooth Muscle Cell Differentiation

For smooth muscle cell formation, LPM cells were dissociated with TrypLE Express (Life Technologies) for 5 minutes at 37ºC, washed once with CDM-PVA and centrifuged at 200g for 3 minutes. Cells were seeded on gelatin and MEF medium coated plates at a density of 2.6×10^4 cells/cm^2 in CDM-PVA supplemented with 10ng/ml PDGF-BB (Peprotech) and 2ng/ml TGF-β (Peprotech) for 12 days. Media was changed every two days and cells split when confluent at a 1:2 ratio, usually on day 3 or day 6 (Cheung et al., 2012).

Cardiomyocyte Differentiation

Following cardiac mesoderm formation, cells were cultured for two days in CDM-BSA (with insulin) supplemented with 8ng/ml FGF2 and 10ng/ml BMP4 (R&D) and subsequently fed every two days with CDM-BSA (with insulin). Onset of beating was observed on day 7-9 of differentiation (Mendjan et al., 2014).

Chondrocyte Differentiation

Following presomitic mesoderm formation, cells were cultured in CDM-BSA (with insulin) supplemented with 8ng/ml FGF2 and 10ng/ml BMP4 (R&D) for 10 days, changing media every two days (Mendjan et al., 2014).

Hepatocyte Differentiation

Protocol for hepatocyte formation has been modified from (Gieseck et al., 2015). Following definitive endoderm formation, cells were cultured for 5 days in RPMI-B27 supplemented with 50ng/ml Activin-A to generate foregut, changing medium daily. Subsequently cells were cultured for 13 days in Hepatozyme supplemented with 20ng/ml OSM (R&D) and 50ng/ml HGF (Peprotech) to generate mature hepatocytes, changing media every two days.

Small Molecule Cell Cycle Inhibitor Treatment

H9 hESCs were treated with the small molecule inhibitors and a number of different doses were tested. The inhibitors used are as follows:

Small Molecule Cell Cycle Inhibitors Used

Inhibitor	Concentrations used	Catalogue Number	Supplier
Aphidicolin	15ng/ml, 75ng/ml	A4487	Sigma-Aldrich
Colcemid	2µg/ml	15212012	Invitrogen
Hydroxyurea	20µM, 100µM, 200µM	H8627	Sigma-Aldrich
L-Mimosine	5µM, 10µM, 50µM	M0253	Sigma-Aldrich
Lovastatin	4µM, 8µM, 16µM	438185	Millipore
Nocodazole	0.02µg/ml, 0.1µg/ml, 0.2µg/ml	M1404	Sigma-Aldrich
Thymidine	400µM, 800µM	T9250	Sigma-Aldrich

Cells were treated with the inhibitors for 16 or 24 hours. For inhibitor removal, cells were washed twice with E8 media and fed normally with maintenance media in the absence of the inhibitors.

Flow Cytometry

For flow cytometry analysis, cells were dissociated into a single cell suspension by incubating with cell dissociation buffer (CDB; Gibco) for 10 minutes at 37ºC and washed once with 1% BSA-PBS. Cells were fixed and permeabilised using the BD Cytofix/Cytoperm solution for 20 minutes at 4ºC (BD Biosciences). After one wash with the perm/wash buffer (BD Biosciences), cells were blocked in perm/wash buffer with 10% donkey serum (Bio-Rad) and 0.1% Triton X-100 (Sigma-Aldrich) for 30 minutes at room temperature. Cells were then stained with the mouse OCT3/4 antibody (C-10; sc5279; Santa Cruz) at 1:200 dilution in perm/wash buffer with 0.1% Triton X-100 for 1 hour at room temperature. Following two washes with the same buffer, cells were incubated with the secondary antibody Alexa Fluor® 647 goat anti-mouse IgM (1:1,000, A21236; Invitrogen) for 1 hour at room temperature in the same buffer, protected from light. Cells were analysed on a Cyan ADP flow cytometer and FlowJo software.

Apoptosis Analysis

Apoptosis was assessed using the Annexin V/Dead Cell Apoptosis kit (Invitrogen) according to the manufacturer's instructions. Briefly, cells were gently dissociated with accutase (Gibco) for 5 min, and washed in E8 media followed by one wash with 1% BSA in PBS. Cells were resuspended in 100 µl 1x binding buffer and mixed with 5 µl of FITC Annexin V and 1 µl propidium iodide for 15 minutes at room temperature. After addition of 400 µl of 1× binding buffer, cells were analysed on a FACS Canto II (BD Biosciences) flow cytometer and FlowJo software.

Western Blot

For protein isolation, cells were washed once with PBS and harvested with cell dissociation buffer (CDB; Gibco) for 10 minutes at 37°C and washed once with cold 1% BSA-PBS. Pellets were collected by centrifugation at 4ºC and 300g for 3 minutes and lysed with RIPA buffer supplemented with protease and phosphatase inhibitors (Roche) for 30 minutes on ice, vortexing every 10 minutes. Following lysis, samples were centrifuged at 4ºC and 17,000g for 5 minutes and the supernatant collected. Protein was quantified using the Pierce BCA Protein Assay Kit (Thermo Fisher Scientific) according to the manufacturer's instructions. Samples were prepared for Western blot analysis by adding 1x NuPAGE LDS Sample Buffer (Thermo Fisher Scientific) and 1% β-mercaptoethanol and boiling the samples at 95ºC for 5 minutes. 10-35 µg of protein was loaded in 4-12% NuPAGE Bis-Tris Precast Gels (Thermo Fisher Scientific) and run using NuPAGE MOPS SDS Running Buffer (Thermo Fisher Scientific). For identification of the size of the target protein, Precision Plus Protein Ladder was used (Bio-Rad). Protein was transferred on PVDF membranes (Bio-Rad) by liquid transfer using NuPAGE Transfer Buffer (Thermo Fisher Scientific). Membranes were blocked using 4% non-fat dried milk in Tris-buffered saline and 0.1% Tween buffer (TBST buffer) for 30 minutes and incubated with primary antibody overnight at 4ºC in TBST. The primary antibodies used are: Cyclin D1 (2922S, Cell Signalling Technology, 1: 1,000), Cyclin D2 (3741S, Cell Signalling Technology, 1: 1,000), Cyclin D3 (2936S, Cell Signalling Technology, 1: 1,000) and α-tubulin (T9026, Sigma-Aldrich, 1: 40,000). Following 3 washes with TBST, membranes were incubated with horseradish peroxidase (HRP)-conjugated secondary antibody for 1 hour at room temperature. Membranes were then washed 3 times with TBST and incubated with Pierce ECL Western Blotting Substrate and exposed to X-Ray Super RX Films (Fujifilm).

RNA Extraction, cDNA Synthesis and qRT-PCR

Total RNA was extracted using the GenElute™ Mammalian Total RNA Miniprep Kit (Sigma-Aldrich) and the On-Column DNase I Digestion set (Sigma-Aldrich) according to the manufacturer's instructions. RNA was reverse transcribed using 250ng random primers (Promega), 0.5mM dNTPs (Promega), 20U RNAseOUT, 0.01M DTT and 25U of SuperScript II (all from Invitrogen). The resulting cDNA was diluted 30-fold for the qPCR

reaction. Quantitative PCR mixtures were prepared using the KAPA SYBR® FAST qPCR Master Mix (2X) Kit (Kapa Biosystems), 4.2µl of cDNA and 200nM of each of the forward and reverse primers. Samples were run on 384 well plates using the QuantStudio 12K Flex Real-Time PCR System machine and results analysed using the delta-delta cycle threshold method (ΔΔCt). Expression values were normalized to the housekeeping gene Porphobilinogen Deaminase (*PBGD*).

qPCR Primers Used

**Gene**	**Forward Primer (5'-3')**	**Reverse Primer (5'-3')**
ACAN	CCCCTGCTATTTCATCGACCC	GACACACGGCTCCACTTGAT
A1AT	CCACCGCCATCTTCTTCCTGCCTGA	GAGCTTCAGGGGTGCCTCCTCTG
ACTN1	CAAACCTGACCGGGGAAAAAT	CTGAATAGCAAAGCGAAGGATGA
ALB	CCTTTGGCACAATGAAGTGGGTAA CC	CAGCAGTCAGCCATTTCACCATAG
CNN1	GTCCACCCTCCTGGCTTT	AAACTTGTTGGTGCCCATCT
COL2A1	TGGACGCCATGAAGGTTTTCT	TGGGAGCCAGATTGTCATCTC
EOMES	ATCATTACGAAACAGGGCAGGC	CGGGGTTGGTATTTGTGTAAGG
NANOG	CATGAGTGTGGATCCAGCTTG	CCTGAATAAGCAGATCCATGG
PAX6	CTTTGCTTGGGAAATCCGAG	AGCCAGGTTGCGAAGAACTC
PBGD	GGAGCCATGTCTGGTAACGG	CCACGCGAATCACTCTCATCT
POU5F1 (OCT4)	AGTGAGAGGCAACCTGGAGA	ACACTCGGACCACATCCTTC
SOX1	Quantitect primers (Qiagen)	Quantitect primers (Qiagen)
SOX17	CGCACGGAATTTGAACAGTA	GGATCAGGGACCTGTCACAC
SOX2	TGGACAGTTACGCGCACAT	CGAGTAGGACATGCTGTAGGT
BRACHYURY	TGCTTCCCTGAGACCCAGTT	GATCACTTCTTTCCTTTGCATCAAG
TAGLN	TCTTTGAAGGCAAAGACATGG	TTATGCTCCTGCGCTTTCTT
TNNT2	ACAGAGCGGAAAAGTGGGAAG	TCGTTGATCCTGTTTCGGAGA

Immunostaining

For immunostaining analysis, cells were fixed for 20 minutes at 4ºC with 4% paraformaldehyde (PFA) in PBS and washed once with PBS. Cells were subsequently blocked and permeabilised at room temperature for 30 minutes in PBS with 4% donkey serum (Bio-Rad) and 0.1% Triton X-100 (Sigma-Aldrich). Primary antibodies were diluted in the same buffer and incubate with the cells for 2 hours at room temperature or overnight at 4ºC. After three washes with PBS, cells were incubated with AlexaFluor secondary antibodies for 1 hour at room temperature protected from light. Cells were subsequently washed three times for 5 minutes with PBS, adding Hoechst 33258 (bis-Benzimide H, 1: 10,000 dilution; Sigma-Aldrich) during the first wash to stain nuclei.

Antibodies Used for Immunostaining Analysis

**Antibody**	**Species**	**Dilution**	**Catalogue Number**	**Manufacturer**
BRACHYURY	Goat	1:200	AF2085	R&D Systems
EOMES	Rabbit	1:500	ab183991	Abcam
HAND1	Goat	1:200	AF3168	R&D Systems
NANOG	Goat	1:200	AF1997	R&D Systems
OCT3/4 (C-10)	Mouse	1:200	sc-5279	Santa Cruz Biotechnology
SOX1	Goat	1:200	AF3369	R&D Systems
SOX17	Goat	1:200	AF1924	R&D Systems
SOX2	Goat	1:200	AF2018	R&D Systems

Alexa Fluor 488 donkey anti-goat	-	1:1,000	A11055	Invitrogen
Alexa Fluor 488 donkey anti-mouse	-	1:1,000	A21202	Invitrogen
Alexa Fluor 488 donkey anti-rabbit	-	1:1,000	A21206	Invitrogen
Alexa Fluor 647 donkey anti-goat	-	1:1,000	A21447	Invitrogen
Alexa Fluor 647 donkey anti-mouse	-	1:1,000	A31571	Invitrogen
Alexa Fluor 647 donkey anti-rabbit	-	1:1,000	A31573	Invitrogen

Alcian Blue Staining of Chondrocytes

Monolayer cultures of chondrocytes were fixed with 4% paraformaldehyde (PFA) for 20 minutes at 4ºC. Cells were then washed with 0.5N HCl and stained overnight with 0.25% (w/v) Alcian Blue 8GX (Sigma-Aldrich) in 0.5N HCl. Stained cells were visualized using a Leica dissecting microscope. Alcian Blue dye was solubilized by overnight incubation with 8M guanidine hydrochloride (Sigma-Aldrich) and quantified by absorbance at 595nm using a spectrophotometer.

SMC Contraction Assay

SMC contraction was induced by treatment with 100µM carbachol (Sigma-Aldrich). Images were acquired with EVOS FL imaging system (Thermo Fisher Scientific) prior to the addition and ten minutes after the addition of carbachol. The surface area of the cells was measured using ImageJ software.

CYP3A4 Assay on Hepatocytes

CYP3A4 activity of the hepatocytes was detected in hepatocytes using the P450-Glo CYP3A4 Assay (Promega) according to the manufacturer's instructions. Luminescence was measured using a GloMax 96 Microplate Luminometer (Promega).

Karyotype and CytoScan Analyses

Cells were treated with 0.1µg/ml colcemid (Gibco) supplemented with 10µM Y27632 (ROCK inhibitor) for 4 hours. Cells were then harvested with cell dissociation buffer (CDB; Gibco) for 10 minutes at 37ºC and centrifuged at 1200rpm for 7 minutes. 0.055M of KCl hypotonic solution was added to the cells and incubated at 37ºC for 30 minutes to allow the cells to swell and release the chromosomes. Following centrifugation at 1200rpm for 7 minutes, cells were fixed with 2ml methanol:glacial acetic acid at a 3:1 ratio. Fixed cell suspensions were sent to the Cytogenetics Laboratory, Cambridge University Hospitals, UK and karyotype analysis was performed by standard G banding techniques to confirm euploidy of the cell lines. For the in-depth CytoScan characterisation, genomic DNA was isolated using the GenElute Mammalian Genomic DNA Miniprep Kit (Sigma-Aldrich). 1µg of genomic DNA was sent to the Cytogenetics Laboratory, Cambridge University Hospitals, UK and analysis was performed using CytoScan 750K Array Kit.

Supplemental References

Cheung, C., Bernardo, A.S., Trotter, M.W.B., Pedersen, R.A., and Sinha, S. (2012). Generation of human vascular smooth muscle subtypes provides insight into embryological origin–dependent disease susceptibility. Nat. Biotechnol. *30*, 165–173.

Gieseck, R.L., Vallier, L., and Hannan, N.R.F. (2015). Generation of Hepatocytes from Pluripotent Stem Cells for Drug Screening and Developmental Modeling. In Protocols in In Vitro Hepatocyte Research, M. Vinken, and V. Rogiers, eds. (New York, NY: Springer New York), pp. 123–142.

Mendjan, S., Mascetti, V.L., Ortmann, D., Ortiz, M., Karjosukarso, D.W., Ng, Y., Moreau, T., and Pedersen, R.A. (2014). NANOG and CDX2 pattern distinct subtypes of human mesoderm during exit from pluripotency. Cell Stem Cell *15*, 310–325.
