# Supplementary material for: Method to Synchronize Cell Cycle of Human Pluripotent Stem Cells without Affecting Their Fundamental Characteristics
Source: Stem Cell Reports. 2018 Dec 27;12(1):165–79. doi: 10.1016/j.stemcr.2018.11.020 (PMC6335580; doi:10.1016/j.stemcr.2018.11.020)
Supplement: Document S2. Article plus Supplemental Information [file mmc2.pdf]

Stem Cell Reports
Article

OPEN ACCESS

Method to Synchronize Cell Cycle of Human Pluripotent Stem Cells without Affecting Their Fundamental Characteristics

Loukia Yiangou,[1,2,3,4,10] Rodrigo A. Grandy,[1,2] Carola M. Morell,[1,2] Rute A. Tomaz,[1,2] Anna Osnato,[1,2] Juned Kadiwala,[5] Daniele Muraro,[4] Jose Garcia-Bernardo,[4] Shota Nakanoh,[1,2,6] William G. Bernard,[1,2] Daniel Ortmann,[1,2] Davis J. McCarthy,[7,8] Ingrid Simonic,[9] Sanjay Sinha,[1,3] and Ludovic Vallier[1,2,4,*]
[1]Wellcome–MRC Cambridge Stem Cell Institute, Anne McLaren Laboratory, University of Cambridge, Cambridge CB2 0SZ, UK
[2]Department of Surgery, University of Cambridge, Cambridge CB2 0QQ, UK
[3]Department of Medicine, Division of Cardiovascular Medicine, University of Cambridge, Cambridge CB2 0QQ, UK
[4]Wellcome Sanger Institute, Wellcome Genome Campus, Hinxton CB10 1SA, UK
[5]Cambridge NIHR Biomedical Research Centre hIPSC Core Facility, University of Cambridge, Cambridge CB2 0SZ, UK
[6]Division of Embryology, National Institute for Basic Biology, Okazaki 444-8787, Japan
[7]European Molecular Biology Laboratory, European Bioinformatics Institute, Wellcome Genome Campus, Hinxton CB10 1SD, UK
[8]St Vincent's Institute of Medical Research, Fitzroy, VIC 3065, Australia
[9]Medical Genetics Laboratories, Cambridge University Hospitals NHS Trust, Cambridge CB2 0QQ, UK
[10]Present address: Department of Anatomy and Embryology, Leiden University Medical Center, Einthovenweg 20, 2333 ZC Leiden, the Netherlands
*Correspondence: lv225@cam.ac.uk
https://doi.org/10.1016/j.stemcr.2018.11.020

SUMMARY

Cell cycle progression and cell fate decisions are closely linked in human pluripotent stem cells (hPSCs). However, the study of these interplays at the molecular level remains challenging due to the lack of efficient methods allowing cell cycle synchronization of large quantities of cells. Here, we screened inhibitors of cell cycle progression and identified nocodazole as the most efficient small molecule to synchronize hPSCs in the G2/M phase. Following nocodazole treatment, hPSCs remain pluripotent, retain a normal karyotype and can successfully differentiate into the three germ layers and functional cell types. Moreover, genome-wide transcriptomic analyses on single cells synchronized for their cell cycle and differentiated toward the endoderm lineage validated our findings and showed that nocodazole treatment has no effect on gene expression during the differentiation process. Thus, our synchronization method provides a robust approach to study cell cycle mechanisms in hPSCs.

INTRODUCTION

Human pluripotent stem cells (hPSCs) represent a unique tool to study early cell fate decisions as they can be grown indefinitely *in vitro* while maintaining the capacity to differentiate into the three germ layers: endoderm, mesoderm, and neuroectoderm (Thomson et al., 1998). The role of the cell cycle machinery in this process has recently been explored and various studies have established that specification of the germ layers is regulated by cell cycle regulators (Pauklin and Vallier, 2013; Pauklin et al., 2016; Singh et al., 2013, 2015); however, extensive biochemical and molecular analyses of these interplays have been hindered by the difficulty of successfully synchronizing a large quantity of stem cells in the different phases of the cell cycle.

Of particular interest, the fluorescence ubiquitination cell cycle indicator (FUCCI) system (Sakaue-Sawano et al., 2008) can be used in hPSCs for live imaging and for sorting cells in different phases of their cell cycle for transcriptomic analyses (Pauklin et al., 2016; Singh et al., 2013). Nonetheless, the FUCCI system presents several limitations. Sorting large amounts of cells is challenging, as it compromises viability and decreases efficacy of differentiation, thereby precluding precise biochemical analyses. In addition, cells in S and G2/M phases cannot be separated using the FUCCI system, limiting studies investigating mechanisms occurring specifically in these phases of the cell cycle. Finally, the FUCCI system does not distinguish between cells in early G1 or quiescence cells. These limitations highlight the need for the development of alternative tools and complementary approaches to synchronize the cell cycle in hPSCs.

Traditionally, somatic cells have been successfully synchronized using small molecules inhibiting cell cycle progression. Those include G1 phase inhibitors, such as lovastatin and mimosine. Lovastatin is a 3-hydroxy-3-methylglutaryl-coenzyme A reductase (HMG-CoA reductase) inhibitor and results in G1 cell cycle arrest by inducing CDKIs, such as p21 and p27 (Hengst et al., 1994; Keyomarsi et al., 1991; Rao et al., 1999). Mimosine is an iron chelator that blocks initiation and elongation of replication forks (Chung et al., 2012; Kalejta and Hamlin, 1997; Krude, 1999; Vacková et al., 2003), resulting in accumulation of cells in the late G1 phase. Inhibitors of G1/S phase transition are also commonly used, such as aphidicolin and thymidine. Thymidine causes inhibition of DNA replication (Thomas and Lingwood, 1975), while aphidicolin blocks DNA polymerase-α, thereby arresting cells at the G1/S phase boundary (Ikegami et al., 1978; Pedrali-Noy et al., 1980). Furthermore, hydroxyurea results in accumulation of cells in the S phase

This is an open access article under the CC BY license (http://creativecommons.org/licenses/by/4.0/).

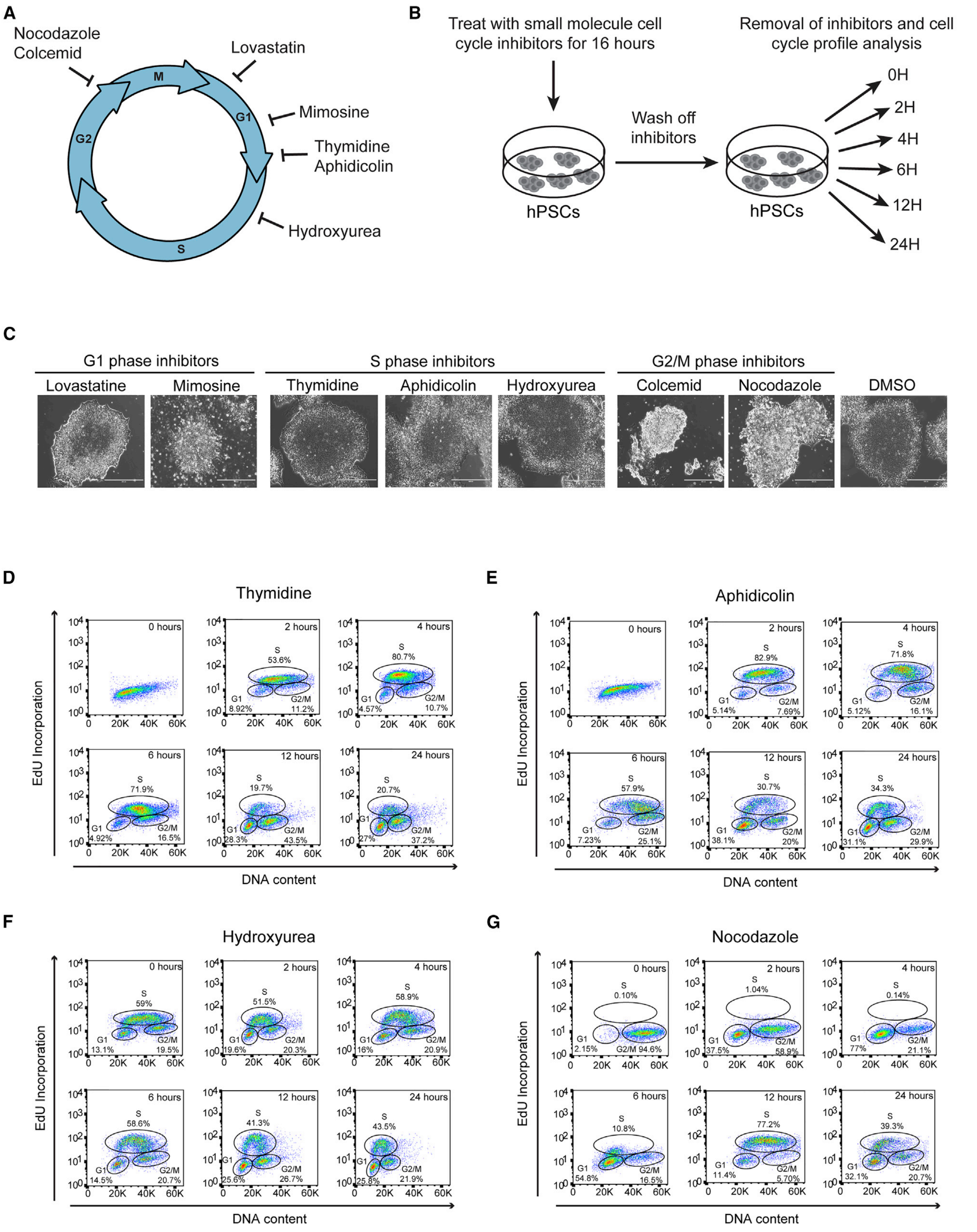

(legend on next page)

by inhibiting ribonucleotide reductase and dNTP production (Adams and Lindsay, 1967; Brigitte Maurer-Schultze and Bassukas, 1988). Last, G2/M phase inhibitors include colcemid and nocodazole. Both inhibit microtubule polymerization and were shown to arrest somatic and embryonic stem cells in G2/M (Blajeski et al., 2002; Grandy et al., 2015). Importantly, previous studies have used some of these molecules to synchronize hPSCs (Calder et al., 2013; Gonzales et al., 2015; Grandy et al., 2015; Yang et al., 2016); however, these methods were often associated with cell death and accumulation of genomic anomalies while their impact on pluripotency and self-renewal remains to be comprehensively analyzed. In this study, we optimized and characterized the use of these inhibitors to synchronize the cell cycle of hPSCs. We observed that a low dose of nocodazole successfully enriches for hPSCs in G2/M without affecting pluripotency and genetic stability. In addition, nocodazole-treated hPSCs can successfully differentiate into the three germ layers and can generate functional cell types, including cardiomyocytes, smooth muscle cells, chondrocytes, and hepatocytes. Finally, we used this approach to differentiate hPSCs into endoderm while being synchronized for their cell cycle, thereby creating an approach to study mechanisms occurring during cell cycle progression upon differentiation. Accordingly, we performed single-cell RNA-sequencing (RNA-seq) analysis during definitive endoderm formation using hPSCs synchronized by nocodazole treatment, and showed that cell cycle synchronization does not affect gene expression or efficiency of differentiation. Taken together, our results demonstrate that cell cycle synchronization by nocodazole does not affect the fundamental characteristics of hPSCs while providing a valuable tool to study the interplays between cell cycle and differentiation.

RESULTS

Nocodazole Is the Only Small Molecule that Can Efficiently Synchronize the Cell Cycle of Human Embryonic Stem Cells

In order to identify small molecules that successfully synchronize human embryonic stem cells (hESCs), we tested a panel of inhibitors commonly used with somatic cell types (Figures 1A and 1B). Conventional doses used in somatic cells resulted in cell death within 6 to 20 hr of treatment (data not shown), indicating that the concentrations of cell cycle inhibitors tolerated by stem cells is different from the threshold tolerated by somatic cells. For this reason, we performed extensive tests to identify the optimal conditions that would block cell cycle progression without toxicity. This screen revealed that only doses up to ten times lower than the ones conventionally used were tolerated by hPSCs. At lower doses, G1 and S phase inhibitors did not affect hPSCs colony morphology with the exception of mimosine, which systematically induced cell death (Figure 1C). Concerning the G2/M inhibitors, most hPSCs were arrested in mitosis and acquired a specific round morphology and increased size (Figure 1C). Having solved the toxicity problem, we then aimed to identify the optimal timing of treatment. For that, we incubated hESCs with each inhibitor for 16 or 24 hr and subsequently performed cell cycle profile analysis using EdU incorporation. Most inhibitors enriched hPSCs in specific cell cycle phases and few differences were observed between the two time-points (Figure S1A). Thus, we decided to apply inhibitors for 16 hr in all subsequent experiments. Concerning the G1 phase inhibitors, lovastatin increased by only 9% the fraction of cells in G1 when compared with DMSO-treated cells. Mimosine treatment resulted in a higher enrichment, with 70% of the cells being in G1 phase; however, most of the cells were dead after 16 hr of treatment (Figure 1C). S phase inhibitors gave different results, with thymidine consistently producing a single population of cells without clear cell cycle phase identity (Figure S1A). This observation can be explained by the fact that cells are blocked at the G1/S transition. Aphidicolin sometimes resulted in the same profile as thymidine, whereas in other cases cells were enriched in the S phase (70%) (Figure S1A), suggesting that the synchronization during G1/S transition is not reliable. Hydroxyurea successfully enriched hPSCs in S phase (70%) while nocodazole treatment successfully enriched hESCs in G2/M (>80%). Colcemid treatment proved less efficient with only around 40% of cells being found in G2/M and was thus excluded from further studies (Figure S1A). Based on these

Figure 1. Nocodazole Is the Most Efficient Small-Molecule Inhibitor to Synchronize the Cell Cycle in hPSCs

(A) Schematic showing the cell cycle phase inhibited by small molecules.

(B) Schematic overview of the experimental setup to determine the efficiency of each small molecule for synchronizing the cell cycle of hESCs.

(C) Brightfield images of colony morphology of H9 hESCs after 16 hr of treatment with the different small molecule cell cycle inhibitors. Scale bars, 400 μm.

(D–G) Cell cycle profile of H9 hESCs following treatment and removal of cell cycle inhibitors thymidine (D), aphidicolin (E), hydroxyurea (F), and nocodazole (G) through a time course of 24 hr.

See also Figure S1.

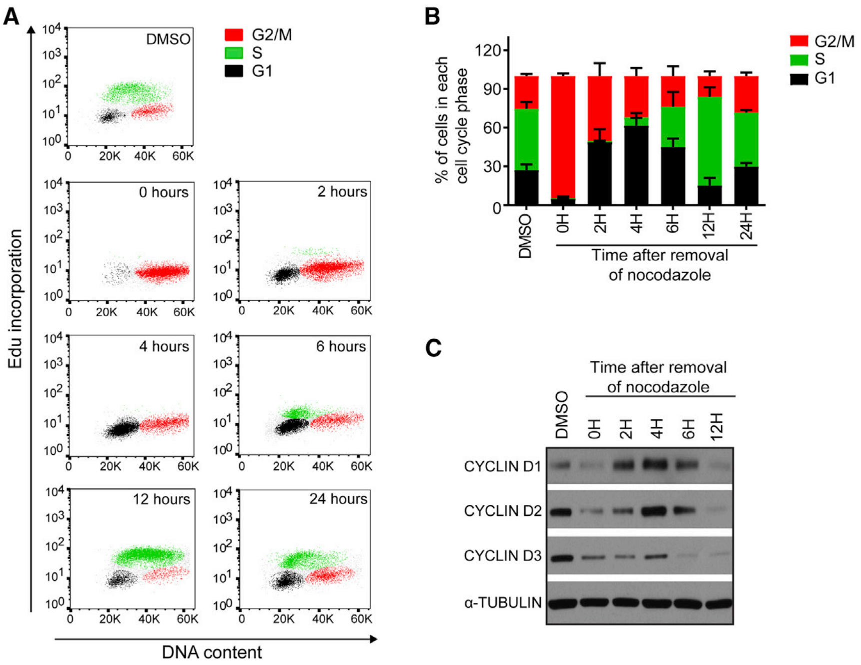

Figure 2. Cell Cycle Synchronization Is Partially Maintained after Release from Nocodazole Inhibition

(A) Cell cycle profile of H9 hESCs following release from nocodazole inhibition. Samples were analyzed through a time course of 24 hr.

(B) Bar graph summarizing the flow cytometry cell cycle profile analysis of H9 hESCs. Error bars represent ±SEM of five independent experiments.

(C) Western blot for cyclin D1, cyclin D2, and cyclin D3 proteins in H9 hESCs through a time course of 24 hr following nocodazole release.

encouraging results, we decided to further refine the optimal dose for each inhibitor. Higher dose systematically improved cell cycle synchronization. However, lovastatin and mimosine treatment still failed to generate homogeneous populations of hESCs blocked in G1 (Figure S1B) and thus were excluded from further studies. Concerning S phase inhibitors, synchronization was very efficient (>70%) (Figure S1B); however, release from these inhibitors systematically resulted in a heterogeneous population. Indeed, removal of thymidine and aphidicolin allowed the cells to progress in S phase (Figures 1D, 1E, S1C, and S1D). However, hESCs became asynchronous 12 hr after release, with 50% of the cells in the G2/M phase upon release from thymidine inhibition, whereas in the case of aphidicolin, cell cycle profile was similar to DMSO-treated cells (Figures 1D, 1E, S1C, and S1D). In the case of hydroxyurea, the percentage of cells in the S phase remained constant throughout the time course after release from inhibition, indicating that the cells remain arrested in the S phase (Figures 1F and S1E). Finally, nocodazole treatment resulted in the most efficient synchronization (>90% of cells in G2/M) while the cells remained synchronous after release and moved homogeneously through the cell cycle for 24 hr. More precisely, the cells progressed into G1 2 hours following removal of nocodazole, with 70% of the cells in G1 at 4 hr and 80% of the cells in S phase after 12 hr (Figures 1G, S1F, 2A, and 2B). Importantly, this synchronization lasted for one cell cycle, after which the cells acquired a heterogeneous cycle profile, thereby suggesting that different hESCs could progress through cell cycle at a different speed (Figures 1G, S1F, 2A and 2B). These observations were confirmed by examining the expression of cyclins D1, D2, and D3, which were specifically enriched in late G1. Accordingly, low levels of cyclin D proteins were observed at time zero after removal of nocodazole when cells were in the G2/M, while their levels steadily increased reaching a peak 4 hr after release when most hESCs are in G1 (Figure 2C). These results demonstrate that nocodazole can be applied to generate a near homogeneous population of hESCs synchronized for their cell cycle without altering cell cycle mechanisms such as periodicity of cell cycle regulators.

hESCs Remain Pluripotent and Karyotypically Normal following Nocodazole Treatment

Importantly, we observed that nocodazole treatment affects morphology of hESC colonies, with the majority of cells increasing in size and losing their epithelial characteristics (Figure 3A). These changes are likely to be associated with the arrest of cell cycle progression in mitosis. Despite the morphological changes observed, treatment with nocodazole did not cause increased apoptosis and cell death, as assessed by Annexin V and propidium iodide analysis (Figures S2A and S2B). Furthermore, nocodazole inhibits microtubule polymerization and this mechanism could result in abnormal chromosome segregation and thus flagrant genetic anomalies. Thus, we decided to investigate whether nocodazole could affect pluripotency and genomic integrity of hESCs. Of note, 12 hr after nocodazole release, the cells recovered and acquired a normal

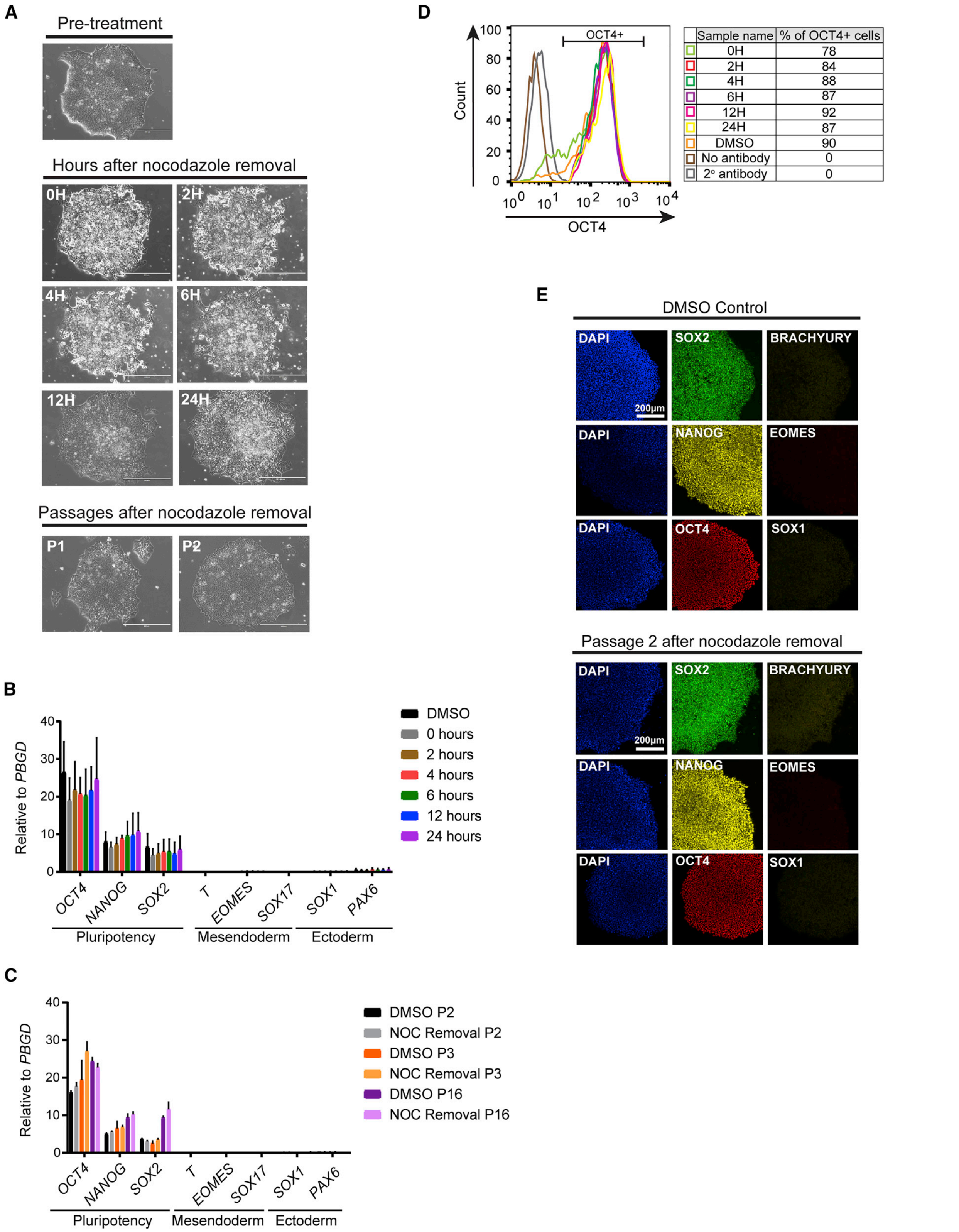

(legend on next page)

morphology, which was maintained over a prolonged period (Figure 3A). In agreement with these observations, gene expression, flow cytometry, and immunostaining analysis showed that expression of *OCT4*, *NANOG*, and *SOX2* were similar in DMSO- and nocodazole-treated cells (Figures 3B–3E). Moreover, absence of markers specific for the three embryonic lineages (*T, EOMES, SOX17, SOX1*, and *PAX6*) confirmed that nocodazole treatment does not cause background differentiation in hESC cultures. These results were confirmed in cells 24 hr after nocodazole release (Figure 3B), as well as passage 2, passage 3, and passage 16 (Figure 3C). Finally, karyotype analyses and extensive investigation for genomic abnormalities using the Affymetrix CytoScan Array did not reveal chromosomal abnormalities in hESCs grown for 10 passages after nocodazole release (Figures S2C and S2D). Considered together, these results confirm that nocodazole treatment does not affect maintenance of pluripotency, does not induce differentiation, and does not compromise the genomic integrity of hESCs even after prolonged periods of time in culture.

Single-Cell RNA-Seq Confirms that Nocodazole Treatment Does Not Affect the Ability of Pluripotent Cells to Differentiate into Definitive Endoderm

To further characterize the effect of cell cycle synchronization, we decided to perform single-cell RNA-seq (scRNA-seq) on nocodazole and DMSO-treated cells before and after differentiation into endoderm. Accordingly, hPSC colonies were treated with DMSO or 100 ng/mL nocodazole for 16 hr and induced to differentiate into definitive endoderm for 3 days. Single cells were subsequently collected in either undifferentiated conditions or after 3 days of endoderm differentiation and then sorted onto 384 well plates for Smart-seq2 processing (Figure 4A). Principal component analysis (PCA) and t-Distributed Stochastic Neighbor Embedding (t-SNE) analysis showed a clear separation between pluripotent and endoderm cells while cell cycle synchronization has no effect on their transcriptional profile with the vast majority of these cells clustering together regardless of their synchronization condition (Figures 4B, S3A, and S3B). Further PCAs show that the main difference between different cell populations (PC1, 40% of variance explained) is their differentiation stage (pluripotent versus endoderm) irrespective of whether they were treated with DMSO or nocodazole (Figures 4B and S3A–S3C). Accordingly, key pluripotency genes, such as *POU5F1* (*OCT4*), *NANOG*, and *SOX2*, were only expressed in pluripotent cells, whereas endoderm genes, such as *SOX17*, *GATA6*, and *CER1*, were expressed in endoderm cells regardless of whether they were treated with DMSO or nocodazole (Figure 4C). These results confirm that nocodazole treatment is compatible with endoderm differentiation.

However, it is important to mention that our analyses also revealed that the endoderm cells could be separated based on their synchronization status (DMSO versus nocodazole). Nonetheless, a more thorough investigation of this dataset showed that this separation is only evident by the third principal component (PC3), which explains less than 6% of the variance among the samples (Figures 4B and S3A–S3C). To confirm this observation, we carried out clustering analysis using a shared nearest neighbor (SNN) modularity optimization algorithm (see Experimental Procedures section). This approach identified five individual clusters that for visualization purposes were presented in a t-SNE plot (Figure 4D). To determine the relationship among these clusters, the average expression for genes in each group was calculated and then used to carry out hierarchical clustering. The top 10 markers of each cluster were selected based on their differential expression when compared with other cells and presented in a heatmap (Figure 4E). This approach revealed that cells coming from the pluripotent cohort were classified in three different clusters. The segregation of clusters 0 and 3 seems to be explained only by biological heterogeneity of the pluripotent population (Figure 4E), whereas cluster 4, based on its proximity to the endoderm cohort, seems to represent a fraction of spontaneously differentiated cells that can be observed in conventional cultures of hPSCs (Figure 4E). Interestingly, these clusters include pluripotent cells both from DMSO and nocodazole conditions, confirming that nocodazole treatment does not affect the fundamental characteristics of pluripotent cells. Concerning endoderm cells, the hierarchical clustering suggests that these two

Figure 3. Nocodazole Treatment Does Not Affect Pluripotency of hESCs

(A) Brightfield images of H9 hESCs showing cell morphology after nocodazole release. Scale bars, 400 μm.

(B) qRT-PCR analysis for pluripotency and differentiation markers in H9 hESCs through a time course of 24 hr after nocodazole release. Error bars represent ±SEM of two independent experiments.

(C) qRT-PCR analysis for pluripotency and differentiation markers in H9 hESCs at passage 2, passage 3, and passage 16 after nocodazole treatment. Error bars represent ±SEM of triplicates in an independent experiment.

(D) Representative flow cytometry analysis for OCT4 expression in H9 hESCs through a time course of 24 hr after nocodazole release.

(E) Immunostaining analysis for the expression of pluripotency markers OCT4, NANOG, and SOX2 and differentiation markers BRACHYURY, EOMES, and SOX1 in DMSO- and nocodazole-treated H9 hESCs, two passages after nocodazole release. Scale bar, 200 μm.

See also Figure S2.

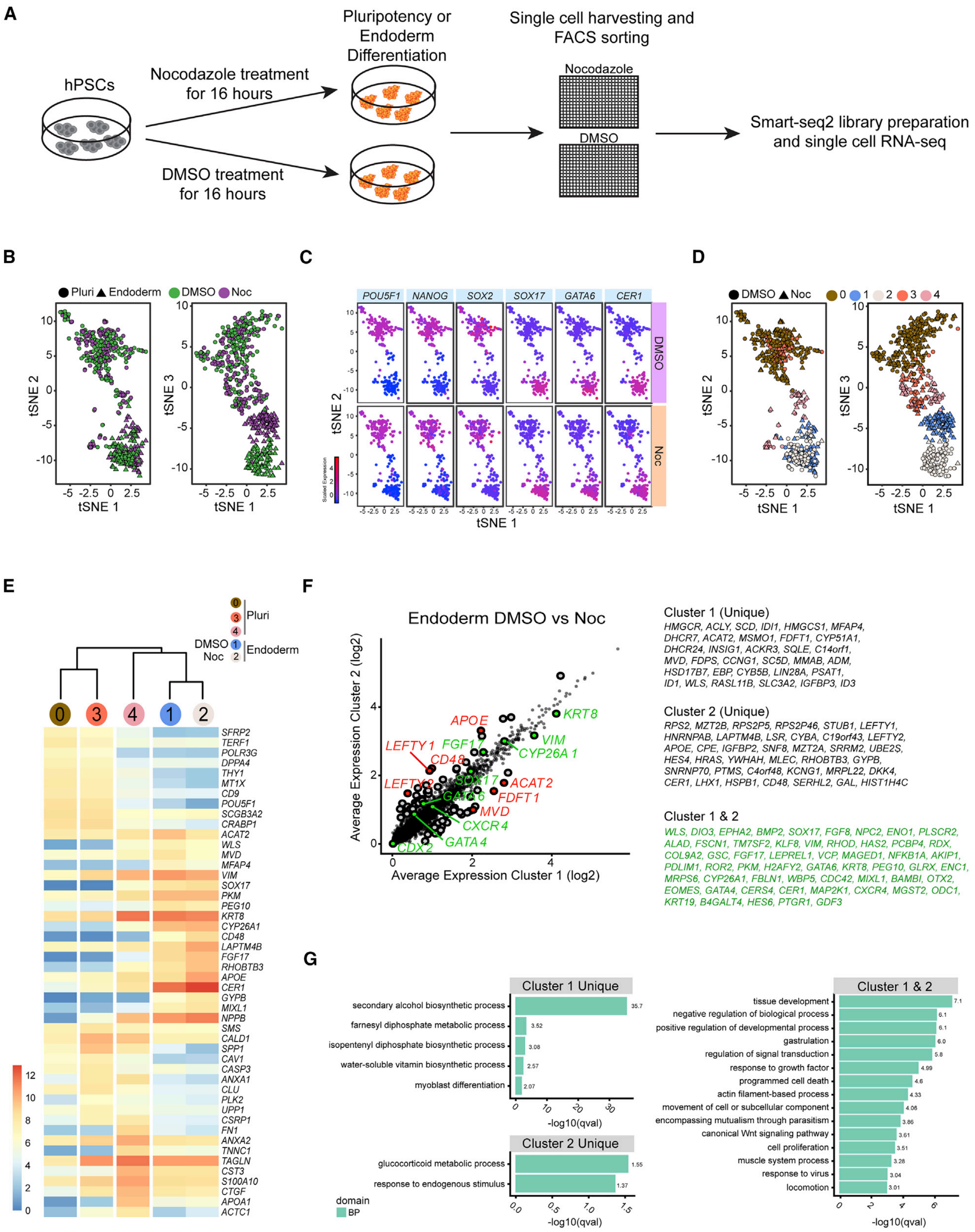

(legend on next page)

groups are highly similar, although our SNN clustering approach did separate the endoderm cohort based on synchronization status (clusters 1 and 2 for DMSO and nocodazole respectively) (Figure 4E). To further confirm this observation, we carried out differential expression analysis for genes in clusters 1 and 2. Accordingly, we found that key endoderm marker genes, such as *SOX17*, *CXCR4*, and *GATA6*, are not among the differentially expressed between these two clusters, indicating that clusters 1 and 2 are similar in terms of differentiation status. However, this approach unveiled 33 genes significantly upregulated in cluster 1 versus cluster 2, and 38 genes significantly upregulated in cluster 2 versus cluster 1 (Figure 4F). The main difference originates from increased expression in genes involved in lipid and cholesterol metabolism (*ACAT2*, *FDFT1*, and *MVD* in cluster 1 and *APOE* in cluster 2, Figure 4F). Gene ontology (GO) analyses for the different clusters further confirmed that differences observed in DMSO- versus nocodazole-treated cells relate to metabolic processes, whereas processes common to both clusters involve tissue development (Figure 4G), thereby confirming the endodermal identity of these cells. The suggested change in metabolic activity could be explained by the lower density systematically observed in nocodazole-treated cells since they undergo at least one cell cycle less than their control. In addition, the loss in epithelial morphology occurring during synchronization in G2/M could also change metabolic requirement in cells treated with nocodazole. In summary, our analyses show that nocodazole synchronization has little effect on the differentiation capacity of the cells into endoderm while it does not affect the cellular identity of undifferentiated pluripotent stem cells or their capacity to differentiate into definitive endoderm.

hESCs Can Successfully Generate All Germ Layers and Functional Cell Types following Nocodazole Treatment

We then decided to further characterize the differentiation capacity of nocodazole-treated hESCs using in-house and previously published protocols for directed differentiation into the three germ layers. H9 hESCs were treated with nocodazole for 16 hr and then grown in culture conditions inducing three mesoderm subtypes (lateral plate mesoderm [LPM], cardiac mesoderm [CM], and presomitic mesoderm [PSM]), as well as endoderm and ectoderm (Figure 5A) (Cheung et al., 2012; Mendjan et al., 2014; Touboul et al., 2010). Immunostaining analysis for early mesoderm markers showed that nocodazole-treated cells differentiated efficiently as seen by the expression of BRACHYURY during LPM (Figure 5B) and PSM induction (Figure 5D) and the expression of EOMES during CM induction (Figure 5C). Moreover, expression of SOX17 during definitive endoderm differentiation was similar between DMSO- and nocodazole-treated cells (Figure 5E), as well as the expression of SOX1 during ectoderm differentiation (Figure 5F).

We further differentiated nocodazole-treated hESCs into functional cell types, such as smooth muscle cells (SMCs), cardiomyocytes, and chondrocytes arising from the mesoderm lineage and hepatocytes arising from the endoderm lineage. Gene expression analyses and functional assays showed a similar level of differentiation efficiency and functionality between DMSO- and nocodazole-treated hESCs. More precisely, SMC production was confirmed by monitoring the expression of *CNN1* and *TAGLN* (Figure 6A) while treatment with the cholinergic agent carbachol resulted in SMC contraction (Figure 6B). Analysis of

Figure 4. Single-Cell RNA-Seq Confirms that Nocodazole Treatment Does Not Affect the Ability of Pluripotent Cells to Differentiate into Definitive Endoderm
(A) Schematic overview of experimental setup for performing single-cell RNA-seq analysis on pluripotent and endoderm cells following nocodazole treatment.
(B) Plots showing two projections of a 3D t-SNE embedding. Dots represent individual cells. Cells were labeled based on their differentiation and synchronization status. Normalized log-expression values were used (DMSO = Green, nocodazole [Noc] = Purple, pluripotent [Pluri] = Circle, Endoderm = Triangle).
(C) t-SNE plot showing the expression pattern of pluripotency (*POUF51*, *NANOG*, *SOX2*) and endoderm (*SOX17*, *GATA6*, *CER1*) genes in each cluster. Dots represent individual cells.
(D) t-SNE plot showing the assignment of clusters identified by applying an SNN modularity optimization algorithm (see Experimental Procedures) in DMSO- and Noc-treated cells. Normalized log-expression values were used. Dots represent individual cells (DMSO = Circle, Noc = Triangle).
(E) Heatmap showing the list of 50 differentially expressed genes obtained when merging the 10 genes with highest average log fold change in each cluster. Clusters 0, 3, and 4 represent undifferentiated cells and clusters 1 and 2 endoderm cells.
(F) Scatterplot showing the log-average expression in cluster 1 versus cluster 2. Genes differentially expressed among cluster 1 and 2 are highlighted in light gray and red, with red representing genes with a log2FC $\geq$ 1. Genes that are not differentially expressed among these two groups are highlighted in green.
(G) GO analyses of clusters 1 and 2 for the genes found in either cluster 1 (cluster 1 unique) or cluster 2 (cluster 2 unique), as well as for those genes that are not differentially expressed between these two groups (clusters 1 and 2).
See also Figure S3.

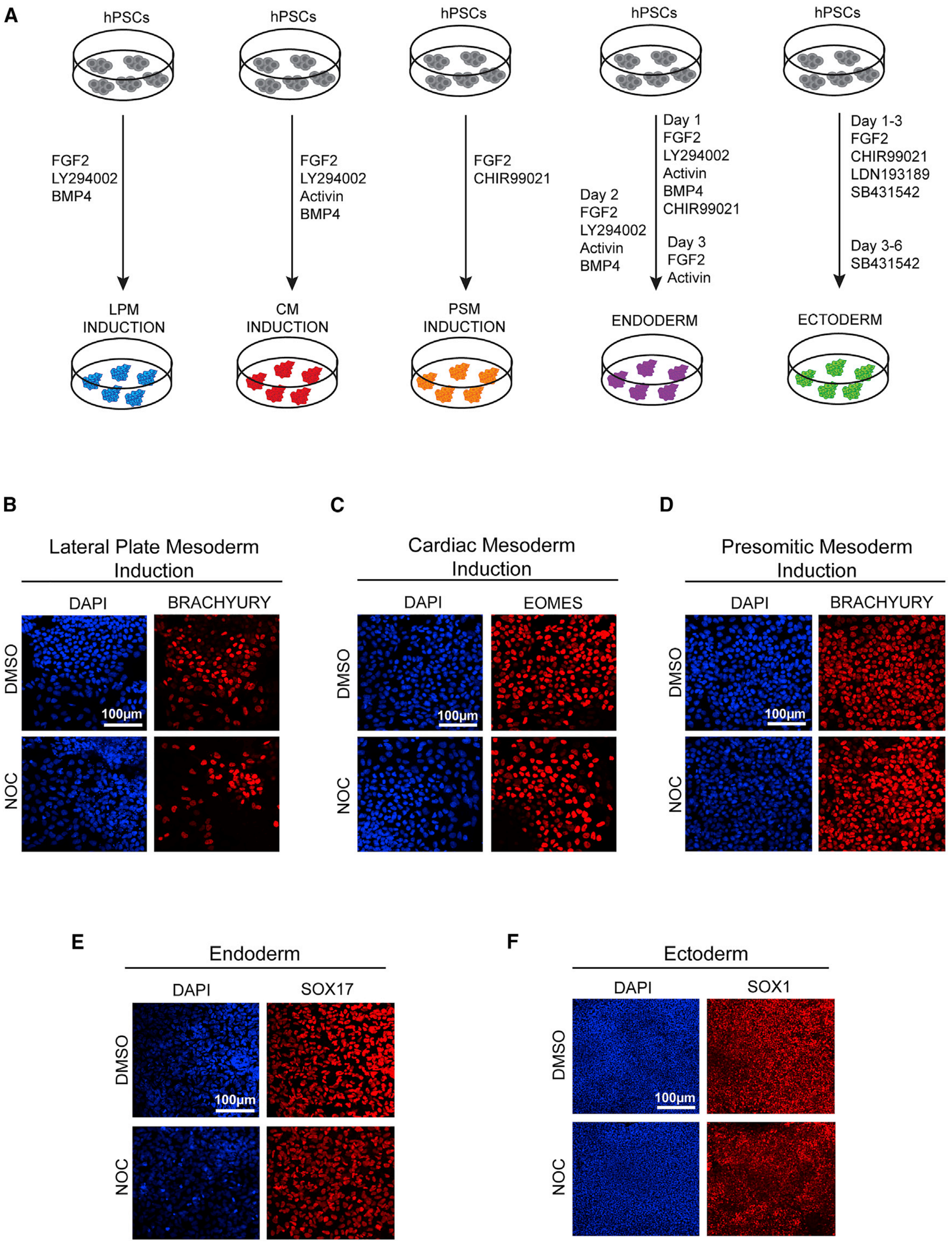

Figure 5. Nocodazole Treatment Does Not Affect the Capacity of hESCs to Differentiate into the Three Germ Layers

(A) Schematic representation of the *in vitro* differentiation protocol to generate the three mesoderm subtypes lateral plate mesoderm (LPM), cardiac mesoderm (CM), and presomitic mesoderm (PSM) as well as endoderm and ectoderm. Treatment for the induction of the three mesoderm subtypes LPM, CM, and PSM is for 36 hr. Treatment for generation of endoderm is for 3 days and for ectoderm 6 days.

(B–F) Immunostaining analysis for BRACHYURY expression during LPM induction (B), EOMES expression during CM induction (C), BRACHYURY expression during PSM induction (D), SOX17 expression in definitive endoderm (E), and SOX1 expression in ectoderm cells (F). Scale bars, 100 μm.

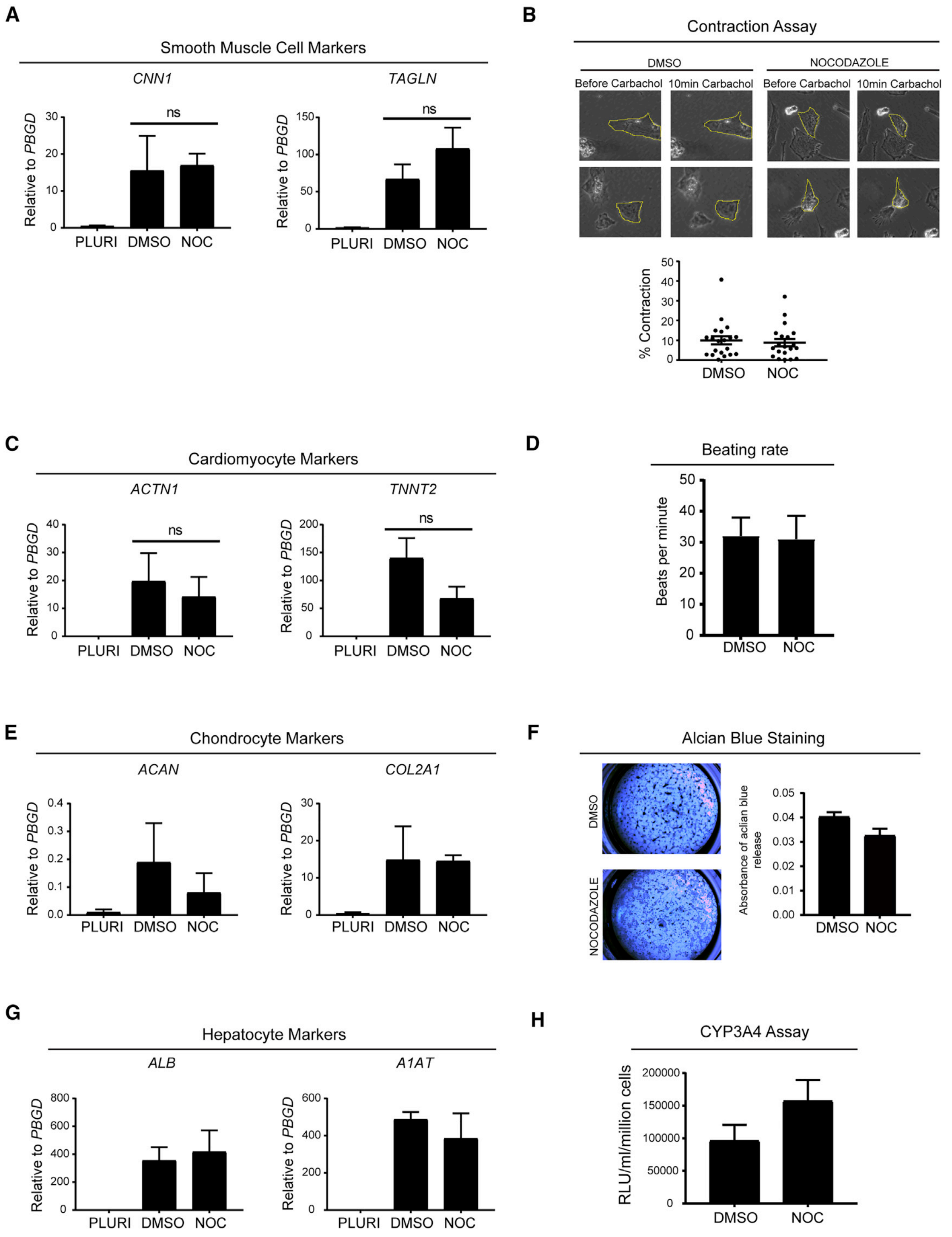

Figure 6. hESCs Can Generate Functional Cell Types following Nocodazole Treatment

(A) qRT-PCR analysis for SMC markers in DMSO and nocodazole-treated cells. Error bars represent ±SEM of three independent experiments. Ordinary one-way ANOVA test followed by Sidak's test for comparison of DMSO versus nocodazole-treated cells was performed. (ns, not significant).

(B) Contractility of SMCs was induced by carbachol. Panels show cells contracting within 10 min of carbachol treatment. Graph shows % contraction of 20 cells in DMSO control and nocodazole-treated cells. Error bars represent ±SEM.

(legend continued on next page)

cardiomyocytes generated from DMSO- and nocodazole-treated cells showed similar levels of expression of the cardiomyocyte markers *ACTN1* and *TNNT2* (Figure 6C), while their beating rate showed no differences (Figure 6D). Similarly, chondrocytes generated from DMSO- and nocodazole-treated cells showed no differences in expression of *ACAN* and *COL2A* (Figure 6E), while functionality was assessed by probing proteoglycan release using Alcian blue staining. Our results showed similar levels of Alcian blue staining and release in DMSO- versus nocodazole-treated chondrocytes (Figure 6F). Concerning endoderm differentiation, DMSO- and nocodazole-treated cells showed high expression of the hepatocyte markers *ALB* and *A1AT* (Figure 6G) and displayed comparable CYP3A4 activity (Figure 6H). In summary, these results show that nocodazole does not affect the capacity of hESCs to differentiate into the three primary germ layers as well as their capacity to produce functional cell types, such as SMCs, cardiomyocytes, chondrocytes, and hepatocytes.

Nocodazole Synchronization Method Works with a Diversity of Human Induced PSCs

To validate that nocodazole synchronization can be applied to a variety of cell lines, we used three additional human induced PSC (hiPSC) lines: a wild-type line (FSPS13B) and two lines derived from patients with cystic fibrosis (CF04 and CF05). These three lines were successfully enriched in the G2/M phase upon nocodazole treatment (Figures 7A–7C), while near homogeneous enrichment in G1 phase (70%) was obtained 4 hr and in S phase (80%) 12 hr after release (Figures 7A–7C). Asynchronous cell cycle profile similar to the DMSO-treated cells was recovered 24 hr after nocodazole release, confirming the results obtained in hESCs (Figures 7A–7C). We then determined the ability of synchronized hiPSC lines to generate mesoderm subtypes. Immunostaining analysis showed that HAND1 was expressed during LPM induction, EOMES during CM induction, and BRACHYURY during PSM induction (Figures 7D–7F). Moreover, karyotypic analyses of three hiPSC lines FSPS13B, CF03, and CF05 7, 16 and 9 passages after nocodazole release respectively, confirmed that the cells maintain a normal karyotype prior to and after treatment with nocodazole (Figures S4A–S4C). In summary, synchronization of cell cycle by nocodazole works efficiently in a diversity of hPSC lines and does not affect their basic characteristics, suggesting that this approach could be used with a broad diversity of cell lines.

DISCUSSION

The limited tools available to study cell cycle dynamics in hPSCs prompted us to characterize and optimize the use of small molecule cell cycle inhibitors to synchronize and enrich hPSCs in the different phases of the cell cycle. A number of reports have shown the use of small molecules, such as nocodazole, aphidicolin, and hydroxyurea, to synchronize hPSCs (Becker et al., 2006; Ghule et al., 2008; Gonzales et al., 2015; Grandy et al., 2015; Neganova et al., 2009; Yang et al., 2016); however, their effect on pluripotency, genetic stability, and capacity of differentiation has not been extensively investigated. In this study, we showed synchronization in S phase using aphidicolin (85% of the cells) and hydroxyurea (70% of the cells) with higher efficiencies than previously reported (Gonzales et al., 2015). Moreover, we reported efficient synchronization by nocodazole with more than 90% of cells enriched in the G2/M phase, while G1 inhibitors were systematically inefficient or toxic to the cells. This last observation suggests that blocking hESCs in this phase of the cell cycle is particularly challenging potentially due its critical function in cell fate decisions (Pauklin and Vallier, 2013; Singh et al., 2013).

Following small molecule treatment and removal, the S phase inhibitors failed to enrich the cells in G2/M and G1 phase, with most of the cells remaining in S phase several hours after release from the inhibitors. This could be due to the inhibitors causing permanent arrest of the

(C) qRT-PCR analysis for cardiomyocyte markers in DMSO and nocodazole-treated cells. Error bars represent ±SEM of three independent experiments. Ordinary one-way ANOVA test followed by Sidak's test for comparison of DMSO versus nocodazole-treated cells was performed (ns, not significant).
(D) Graph showing beating rate of cardiomyocytes generated form DMSO and nocodazole-treated cells. Error bars represent ±SEM (n = 4).
(E) qRT-PCR analysis for chondrocyte markers in DMSO- and nocodazole-treated cells. Error bars represent ±SEM of two independent experiments.
(F) Alcian blue staining of chondrocytes shows Alcian blue absorption and release of DMSO control and nocodazole-treated cells. Error bars represent ±SEM of triplicates in an independent experiment.
(G) qRT-PCR analysis for hepatocyte markers in DMSO and nocodazole-treated cells. Error bars represent ±SEM of triplicates in an independent experiment.
(H) Hepatocytes generated from DMSO and nocodazole-treated cells display cytochrome P450 3A4 activity, as assessed by the enzymatic release of free luciferin by cytochrome P450 from an inactive luciferin precursor. Error bars represent ±SEM of triplicates in an independent experiment.

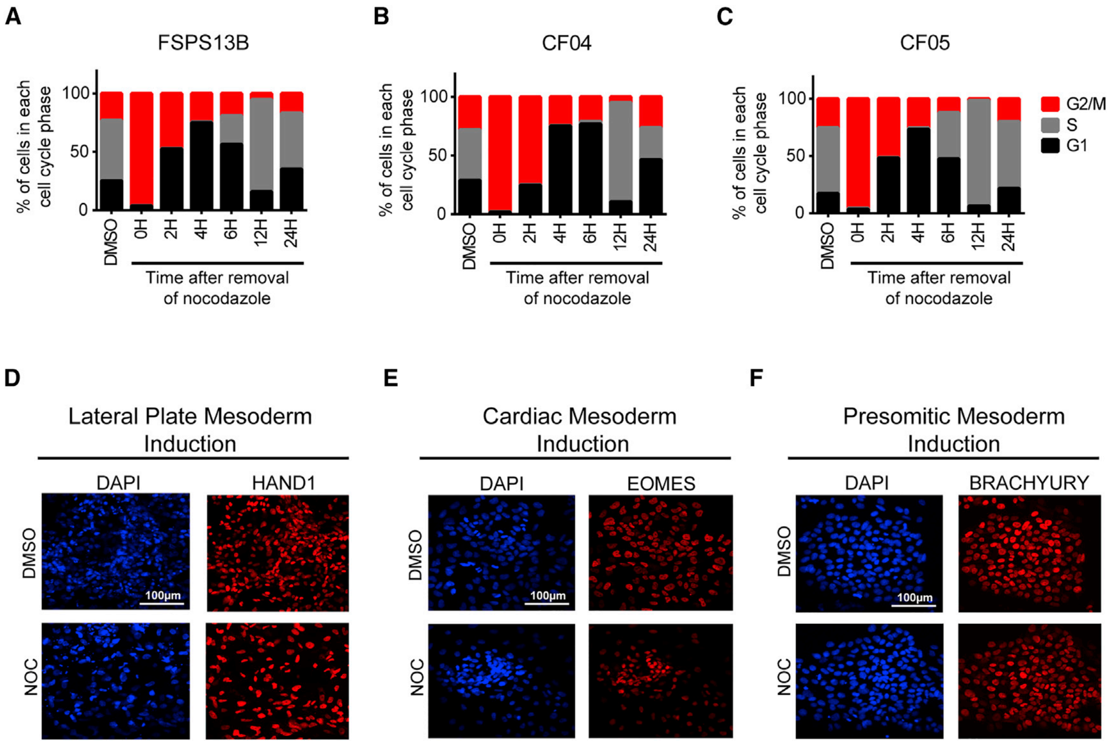

Figure 7. Human iPSCs Can Be Synchronized with Nocodazole while Maintaining Their Capacity of Differentiation
(A–C) Cell cycle profile of hiPSCs lines FSPS13B (A), CF04 (B), and CF05 (C) following treatment and release from nocodazole.
(D–F) Immunostaining analysis for the expression of early mesoderm markers HAND1 (during LPM induction, D), EOMES (during CM induction, E), and BRACHYURY (during PSM induction, F) in DMSO- and nocodazole-treated cells after 36 hr of differentiation. Scale bar, 100 μm.
See also Figure S4.

cells. Furthermore, S phase lasts more than 6 hr in hPSCs and thus synchronization is unlikely to be homogeneous. The G2/M inhibitor nocodazole proved to be the most successful inhibitor not only in blocking cell cycle progression but also in producing populations of hPSCs synchronous for cell cycle progression after release without causing significant cell death. We also showed that the expression of cyclin D proteins elicit the expected periodicity in the different cell cycle phases, suggesting that nocodazole treatment does not perturb the cell cycle machinery. Of note, synchronization was maintained in part for one cell cycle after release (70% cells in G1 and 80% cells in S phase) suggesting that our approach could be useful to study events happening in each of these cell cycle phases. However, investigating mechanisms occurring in a very precise and time-limited phase of the cell cycle such as early G1 or G1/S transition, might require additional sorting strategy.

The efficiency of nocodazole synchronization could be explained by its effect on microtubule polymerization during mitosis, which represents a very short phase of the cell cycle in hPSCs. Thus, blocking cell cycle progression in this phase of the cell cycle would result in a homogeneous and synchronized population. Further characterization showed that nocodazole treatment did not affect pluripotency in agreement with previous reports (Grandy et al., 2015; Yang et al., 2016). Of note, a previous report stated that expression of pluripotency markers is reduced irreversibly upon nocodazole treatment. Nonetheless, this report did not use the same protocol of synchronization and successful enrichment in the G2/M phase upon nocodazole treatment was not observed (approximately 53%). Thus, different dose/time and culture conditions are likely to affect the efficacy of nocodazole treatment and its effect on pluripotency (Kallas et al., 2011).

We further used our approach to perform single-cell RNA-seq analysis of synchronous and asynchronous cells during the process of endoderm differentiation. These analyses showed that nocodazole-treated hPSCs efficiently differentiated into a near homogeneous population of endoderm cells after 72 hr. Nonetheless, nocodazole treatment increased the heterogeneity of the endoderm population probably by decreasing the speed by which some cells

can reach the endoderm state. This delay could be explained by the lower density systematically observed in nocodazole-treated cells that undergo at least one cell cycle less than their DMSO-treated counterpart. In addition, the loss in epithelial morphology observed during nocodazole treatment could decrease the speed by which nocodazole-treated cells can differentiate. Finally, the start of the differentiation and/or the exit from pluripotency could be delayed by the inhibition of cell cycle progression as suggested by others (Gonzales et al., 2015).

Importantly, this increase in heterogeneity had little or no effect on the capacity of differentiation of hPSCs or on the production of terminally differentiated cell types. Indeed, nocodazole-treated cells were able to efficiently generate all the germ layers and some of their derivatives, including SMCs, cardiomyocytes, chondrocytes, and hepatocytes. These findings were validated on three independent hiPSC lines, thereby demonstrating the robustness of our method for synchronizing cell cycle in pluripotent stem cells. Thus, cell synchronization of hPSCs by nocodazole does not affect their fundamental properties.

To conclude, the approach described in our study will enable new investigations, especially detailed molecular analyses of the interplays between cell cycle machinery, transcription factors, and epigenetic modifiers during cell cycle progression in pluripotent stem cells and during differentiation.

EXPERIMENTAL PROCEDURES

hPSC Culture and Differentiation

H9 hESCs (WiCell, Madison, WI, USA) and the hiPSC lines FSPS13B, CF03, CF04, and CF05 were plated on vitronectin-coated plates (10 μg/mL, Stem Cell Technologies) and cultured in E6 media supplemented with 2 ng/mL transforming growth factor β (R&D) and 25 ng/mL fibroblast growth factor 2 (Dr. Marko Hyvönen, Cambridge University) making complete E8 media. Cells were maintained by weekly passaging using 0.5 mM EDTA (Thermo Fisher Scientific). The cells were differentiated into the three germ layers and functional cell types as previously described (Cheung et al., 2012; Mendjan et al., 2014) and as described in the Supplemental Information.

Synchronization and Differentiation of Cells Using Nocodazole

For synchronization into the G2/M phase of the cell cycle, cells were treated with 100 ng/mL of nocodazole (Sigma-Aldrich) for 16 hr. For enrichment of the cells into the different cell cycle phases, cells were washed twice with E8 media and cultured in maintenance media for 2, 4, 6, 12, and 24 hr. For differentiation following nocodazole treatment, cells were plated and treated with nocodazole the next day (for mesoderm differentiation) or 2–3 days after plating (for endoderm and ectoderm differentiation). Following two washes with E8 media, cells were induced to differentiate using the protocols described in the Supplemental Information.

Cell Cycle Profile Analysis

Cell cycle profile analysis was performed using the Click-iT EdU Pacific Blue Flow Cytometry Assay Kit (Thermo Fisher Scientific) according to the manufacturer's instructions. In summary, cultured cells were incubated at 37°C with 10 μM EdU (5-ethynyl-2′-deoxyuridine) for 1 hr and harvested using cell dissociation buffer (Gibco). After three washes with PBS/1% BSA, cells were fixed with 4% paraformaldehyde for 15 min at room temperature and washed three more times with PBS/1% BSA. Cells were then permeabilized for 15 min with saponin-based permeabilization/wash buffer and incubated with the Click-iT reaction cocktail for 30 min protected from light. Cells were washed once with saponin-based permeabilization/wash buffer and stained for DNA content using the FxCycle Far Red dye (Invitrogen). Cells were analyzed on the Cyan ADP flow cytometer and FlowJo software.

Single-Cell RNA-Seq

hiPSCs (FSPS13B) were either treated with DMSO or nocodazole 16 hr before the start of differentiation. Cell sorting and library preparation was carried out by the sequencing core facility at the Sanger Institute. Briefly, single hPSCs were isolated into 384-well plates and libraries were prepared for 120 cells per condition using the Smart-seq2 protocol. A constant amount of spike-in RNA from the External RNA Controls Consortium was also added to the lysis buffer prior to sorting. Transcript expression quantification was performed with "Salmon" (Patro et al., 2017) and collapsed to gene level using "Scater" (McCarthy et al., 2017); Quality control metrics calculations, normalization, and PCA analyses were carried out using "Scater" and "Seurat" (Butler et al., 2018). Low-quality cells were removed based on total number of counts/cell, proportion of counts in mitochondrial genes, or spike-in transcripts. Normalization was performed in Seurat by applying the default "LogNormalize" method that normalizes the gene expression measurements for each cell by the total expression, multiplies this by a scale factor and log-transforms the result. Highly variable genes were selected, the number of detected molecules was regressed out, and scaled *Z*-scored residuals were employed for downstream analysis. An SNN modularity optimization-based algorithm (Waltman and Van Eck, 2013) was used to identify the clusters presented in Figure 4D. Markers for every cluster were identified by calculating differential expression of each cluster compared with all remaining cells (Wilcoxon rank-sum test). The 10 genes with highest average log fold change were selected for each cluster as top markers and their log-average expression was used as an input for hierarchical clustering (Figure 4E). Enrichment of GO biological processes was obtained with the R package g:Profiler (Reimand et al., 2007). Due to the hierarchical structure of GO terms, the categories were grouped together when sharing enriched parents by applying the "moderate" option, which selects the most significant category from each of such groups. Correction for multiple testing was performed with the false discovery rate. RNA-seq data have been deposited in the ArrayExpress database at EMBL-EBI (www.ebi.ac.uk/arrayexpress) under accession number E-MTAB-7008.

Statistical Analysis

Statistical analyses were performed using GraphPad Prism 7 software. The type of statistical analysis performed in each experiment and the number of replicates used are described in the figure legends. For comparison of multiple groups, one-way ANOVA was performed. Significance in each analysis is represented by *p < 0.05, **p < 0.01, ***p < 0.001, ****p < 0.0001, ns = not significant.

SUPPLEMENTAL INFORMATION

Supplemental Information includes Supplemental Experimental Procedures and four figures and can be found with this article online at https://doi.org/10.1016/j.stemcr.2018.11.020.

AUTHOR CONTRIBUTIONS

L.Y. designed, performed, and analyzed experiments, and wrote the manuscript. R.A.G. designed, performed and analyzed experiments. C.M.M. and R.A.T. assisted with hESC differentiations into mature hepatocytes. A.O. assisted with experimental work. J.K. assisted with preparation of hPSCs for karyotyping. D.M. assisted with single-cell RNA-seq analysis and interpretation. J.G.-B. assisted with design, organization, and analysis of the single-cell RNA-seq experiment. S.N. assisted with preparation of hPSCs for karyotyping. W.G.B. assisted with the SMC contraction and apoptosis assays. D.O. assisted with the apoptosis assay. D.J.M. assisted with single-cell RNA-seq analysis. I.S. assisted with karyotyping and CytoScan array analyses. S.S. supervised and supported the study. L.V. conceived, supervised, and supported the study, and wrote and gave final approval to the manuscript.

ACKNOWLEDGMENTS

We thank the Cytometry Core Facility (CCR) at the Wellcome Sanger Institute for performing the single cell sort and the Cytogenetics Laboratory, Cambridge University Hospitals, UK for karyotyping and CytoScan array analyses. This work was supported by the Wellcome PhD program (PSAG/048 to L.Y. and PSAG/051 to A.O.); the European Research Council advanced grant New-Chol (ERC: 741707 to L.V. and R.A.G.), the Cambridge University Hospitals National Institute for Health Research Biomedical Research Center (to L.V.); an NC3Rs grant (NC/N001540/1 to C.M.M.), an MRC UK-RPM II grant (to R.A.T.), a Grant-in-Aid for JSPS Research Fellow (16J08005 to S.N.), a BHF Senior Research Fellowship (FS/13/29/30024 to S.S.), the Cystic Fibrosis Foundation, the Cystic Fibrosis Trust, a core support grant from the Wellcome and Medical Research Council to the Wellcome–Medical Research Council Cambridge Stem Cell Institute (PSAG028), and a core support grant from the Wellcome to the Wellcome Sanger Institute (WT206194).

Received: July 3, 2018
Revised: November 28, 2018
Accepted: November 29, 2018
Published: December 27, 2018

REFERENCES

Adams, R.L.P., and Lindsay, J.G. (1967). Hydroxyurea reversal of inhibition and use as a cell-synchronizing agent. J. Biol. Chem. *242*, 1314–1317.

Becker, K.A., Ghule, P.N., Therrien, J.A., Lian, J.B., Stein, J.L., van Wijnen, A.J., and Stein, A.G.S. (2006). Self-renewal of human embryonic stem cells is supported by a shortened G1 cell cycle phase. J. Cell. Physiol. *209*, 883–893.

Blajeski, A.L., Phan, V.A., Kottke, T.J., and Kaufmann, S.H. (2002). G1 and G2 cell-cycle arrest following microtubule depolymerization in human breast cancer cells. J. Clin. Invest. *110*, 91–99.

Brigitte Maurer-Schultze, M.S., and Bassukas, I.D. (1988). An in vivo study on the synchronizing effect of hydroxyurea. Exp. Cell Res. *174*, 230–243.

Butler, A., Hoffman, P., Smibert, P., Papalexi, E., and Satija, R. (2018). Integrating single-cell transcriptomic data across different conditions, technologies, and species. Nat. Biotechnol. *36*, 411–420.

Calder, A., Roth-Albin, I., Bhatia, S., Pilquil, C., Lee, J.H., Bhatia, M., Levadoux-Martin, M., McNicol, J., Russell, J., Collins, T., et al. (2013). Lengthened G1 phase indicates differentiation status in human embryonic stem cells. Stem Cells Dev. *22*, 279–295.

Cheung, C., Bernardo, A.S., Trotter, M.W.B., Pedersen, R.A., and Sinha, S. (2012). Generation of human vascular smooth muscle subtypes provides insight into embryological origin-dependent disease susceptibility. Nat. Biotechnol. *30*, 165–173.

Chung, L.-C., Tsui, K.-H., Feng, T.-H., Lee, S.-L., Chang, P.-L., and Juang, H.-H. (2012). L-Mimosine blocks cell proliferation via upregulation of B-cell translocation gene 2 and N-myc downstream regulated gene 1 in prostate carcinoma cells. Am. J. Physiol. Cell Physiol. *302*, C676–C685.

Ghule, P.N., Dominski, Z., Yang, X.-C., Marzluff, W.F., Becker, K.A., Harper, J.W., Lian, J.B., Stein, J.L., van Wijnen, A.J., and Stein, G.S. (2008). Staged assembly of histone gene expression machinery at subnuclear foci in the abbreviated cell cycle of human embryonic stem cells. Proc. Natl. Acad. Sci. U S A *105*, 16964–16969.

Gonzales, K.A.U., Liang, H., Lim, Y.-S., Chan, Y.-S., Yeo, J.-C., Tan, C.-P., Gao, B., Le, B., Tan, Z.-Y., Low, K.-Y., et al. (2015). Deterministic restriction on pluripotent state dissolution by cell-cycle pathways. Cell *162*, 564–579.

Grandy, R.A., Whitfield, T.W., Wu, H., Fitzgerald, M.P., VanOudenhove, J.J., Zaidi, S.K., Montecino, M.A., Lian, J.B., VanWijnen, A.J., Stein, J.L., et al. (2015). Genome-wide studies reveal that h3k4me3 modification in bivalent genes is dynamically regulated during the pluripotent cell cycle and stabilized upon differentiation. Mol. Cell. Biol. *36*, 615–627.

Hengst, L., Dulic, V., Slingerland, J.M., Lees, E., and Reed, S.I. (1994). A cell cycle-regulated inhibitor of cyclin-dependent kinases. Proc. Natl. Acad. Sci. U S A *91*, 5291–5295.

Ikegami, S., Taguchi, T., Ohashi, M., Oguro, M., Nagano, H., and Mano, Y. (1978). Aphidicolin prevents mitotic cell division by interfering with the activity of DNA polymerase-alpha. Nature *275*, 458–460.

Kalejta, R.F., and Hamlin, J.L. (1997). The dual effect of mimosine on DNA replication. Exp. Cell Res. *183*, 173–183.

Kallas, A., Pook, M., Maimets, M., Zimmermann, K., and Maimets, T. (2011). Nocodazole treatment decreases expression of pluripotency markers nanog and Oct4 in human embryonic stem cells. PLoS One *6*, e19114.

Keyomarsi, K., Sandoval, L., Band, V., and Pardee, A. (1991). Synchronization of tumor and normal cells from g 1 to multiple cell cycles by lovastatin. Cancer Res. *51*, 3602–3609.

Krude, T. (1999). Mimosine arrests proliferating human cells before onset of DNA replication in a dose-dependent manner. Exp. Cell Res. *247*, 148–159.

McCarthy, D.J., Campbell, K.R., Lun, A.T.L., and Wills, Q.F. (2017). Scater: pre-processing, quality control, normalization and visualization of single-cell RNA-seq data in R. Bioinformatics *33*, 1179–1186.

Mendjan, S., Mascetti, V.L., Ortmann, D., Ortiz, M., Karjosukarso, D.W., Ng, Y., Moreau, T., and Pedersen, R.A. (2014). NANOG and CDX2 pattern distinct subtypes of human mesoderm during exit from pluripotency. Cell Stem Cell *15*, 310–325.

Neganova, I., Zhang, X., Atkinson, S., and Lako, M. (2009). Expression and functional analysis of G1 to S regulatory components reveals an important role for CDK2 in cell cycle regulation in human embryonic stem cells. Oncogene *28*, 20–30.

Patro, R., Duggal, G., Love, M.I., Irizarry, R.A., and Kingsford, C. (2017). Salmon provides fast and bias-aware quantification of transcript expression. Nat. Methods *14*, 417–419.

Pauklin, S., and Vallier, L. (2013). The cell-cycle state of stem cells determines cell fate propensity. Cell *155*, 135–147.

Pauklin, S., Madrigal, P., Bertero, A., and Vallier, L. (2016). Initiation of stem cell differentiation involves cell cycle-dependent regulation of developmental genes by Cyclin D. Genes Dev. *30*, 421–433.

Pedrali-Noy, G., Spadari, S., Miller-Faurès, A., Miller, A.O., Kruppa, J., and Koch, G. (1980). Synchronization of HeLa cell cultures by inhibition of DNA polymerase alpha with aphidicolin. Nucleic Acids Res. *8*, 377–387.

Rao, S., Porter, D.C., Chen, X., Herliczek, T., Lowe, M., and Keyomarsi, K. (1999). Lovastatin-mediated G1 arrest is through inhibition of the proteasome, independent of hydroxymethyl glutaryl-CoA reductase. Proc. Natl. Acad. Sci. U S A *96*, 7797–7802.

Reimand, J., Kull, M., Peterson, H., Hansen, J., and Vilo, J. (2007). G: profiler—a web-based toolset for functional profiling of gene lists from large-scale experiments. Nucleic Acids Res. *35*, 193–200.

Sakaue-Sawano, A., Kurokawa, H., Morimura, T., Hanyu, A., Hama, H., Osawa, H., Kashiwagi, S., Fukami, K., Miyata, T., Miyoshi, H., et al. (2008). Visualizing spatiotemporal dynamics of multicellular cell-cycle progression. Cell *132*, 487–498.

Singh, A.M., Chappell, J., Trost, R., Lin, L., Wang, T., Tang, J., Wu, H., Zhao, S., Jin, P., and Dalton, S. (2013). Cell-cycle control of developmentally regulated transcription factors accounts for heterogeneity in human pluripotent cells. Stem Cell Reports *1*, 532–544.

Singh, A.M., Sun, Y., Li, L., Zhang, W., Wu, T., Zhao, S., Qin, Z., and Dalton, S. (2015). Cell-cycle control of bivalent epigenetic domains regulates the exit from pluripotency. Stem Cell Reports *5*, 1–14.

Thomas, D.B., and Lingwood, C.A. (1975). A model of cell cycle control: effects of thymidine on synchronous cell cultures. Cell *5*, 37–42.

Thomson, J.A., Itskovitz-Eldor, J., Shapiro, S.S., Waknitz, M.A., Swiergiel, J.J., Marshall, V.S., and Jones, J.M. (1998). Embryonic stem cell lines derived from human blastocysts. Science *282*, 1145–1148.

Touboul, T., Hannan, N.R.F., Corbineau, S., Martinez, A., Martinet, C., Branchereau, S., Mainot, S., Strick-Marchand, H., Pedersen, R., Di Santo, J., et al. (2010). Generation of functional hepatocytes from human embryonic stem cells under chemically defined conditions that recapitulate liver development. Hepatology *51*, 1754–1765.

Vacková, I., Engelová, M., Marinov, I., and Tománek, M. (2003). Cell cycle synchronization of porcine granulosa cells in G1 stage with mimosine. Anim. Reprod. Sci. *77*, 235–245.

Waltman, L., and Van Eck, N.J. (2013). A smart local moving algorithm for large-scale modularity-based community detection. Eur. Phys. J. B *86*, 471.

Yang, D., Scavuzzo, M.A., Chmielowiec, J., Sharp, R., Bajic, A., and Borowiak, M. (2016). Enrichment of G2/M cell cycle phase in human pluripotent stem cells enhances HDR-mediated gene repair with customizable endonucleases. Sci. Rep. *6*, 21264.

Stem Cell Reports, Volume *12*

Supplemental Information

Method to Synchronize Cell Cycle of Human Pluripotent Stem Cells without Affecting Their Fundamental Characteristics

Loukia Yiangou, Rodrigo A. Grandy, Carola M. Morell, Rute A. Tomaz, Anna Osnato, Juned Kadiwala, Daniele Muraro, Jose Garcia-Bernardo, Shota Nakanoh, William G. Bernard, Daniel Ortmann, Davis J. McCarthy, Ingrid Simonic, Sanjay Sinha, and Ludovic Vallier

SUPPLEMENTAL INFORMATION

Supplemental Figures

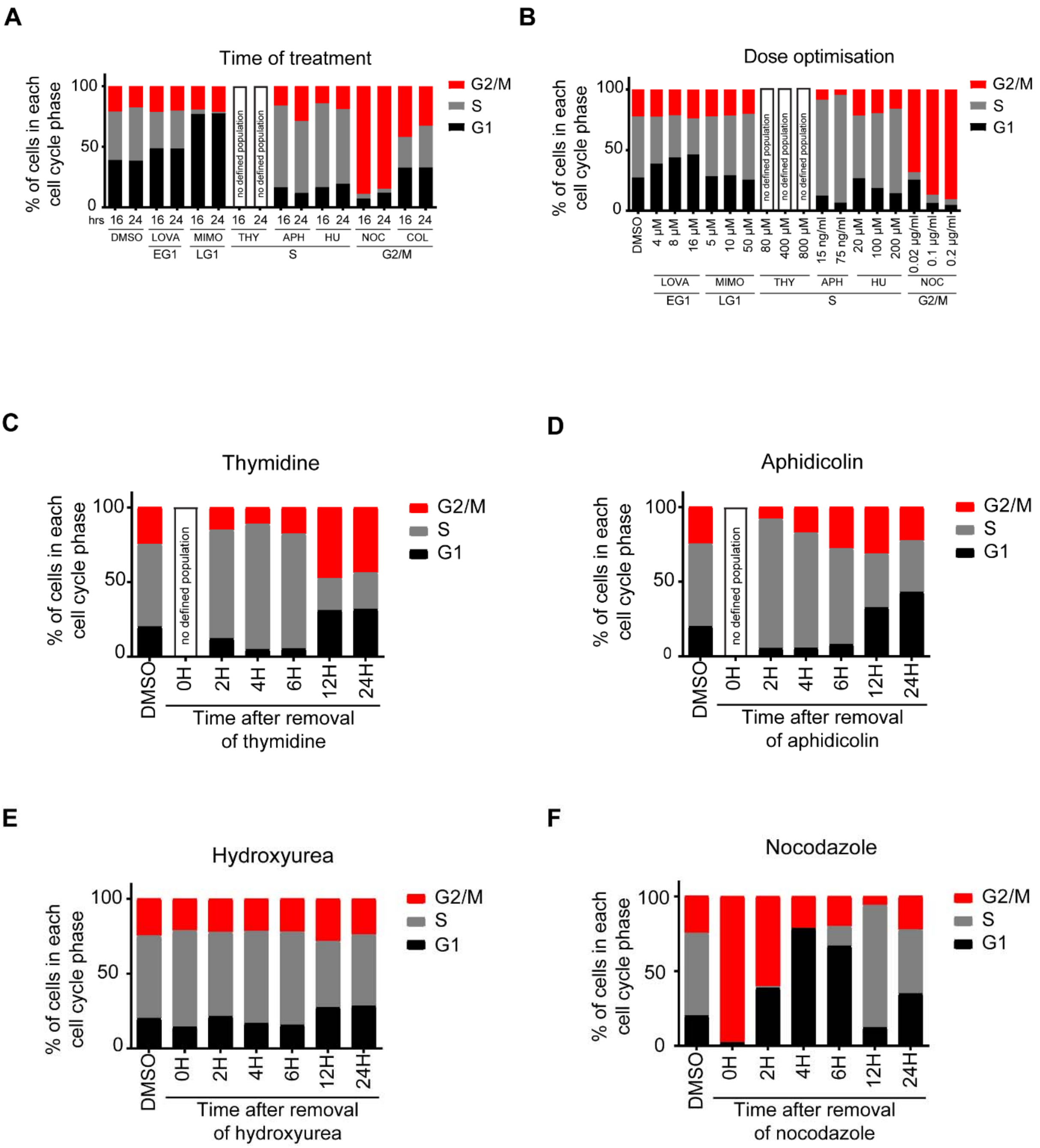

Figure S1. Optimisation of timing and dose of small molecule cell cycle inhibitor treatment. Related to Figure 1.

(A) Cell cycle profile of H9 hESCs, incubated for 16 or 24 hours with the small molecule cell cycle inhibitors.

(B) Cell cycle profile of H9 hESCs, incubated with different doses of the small molecule cell cycle inhibitors.

(C-F) Cell cycle profile of H9 hESCs following treatment and removal of cell cycle inhibitors thymidine (C), aphidicolin (D), hydroxyurea (E) and nocodazole (F) through a timecourse of 24 hours.

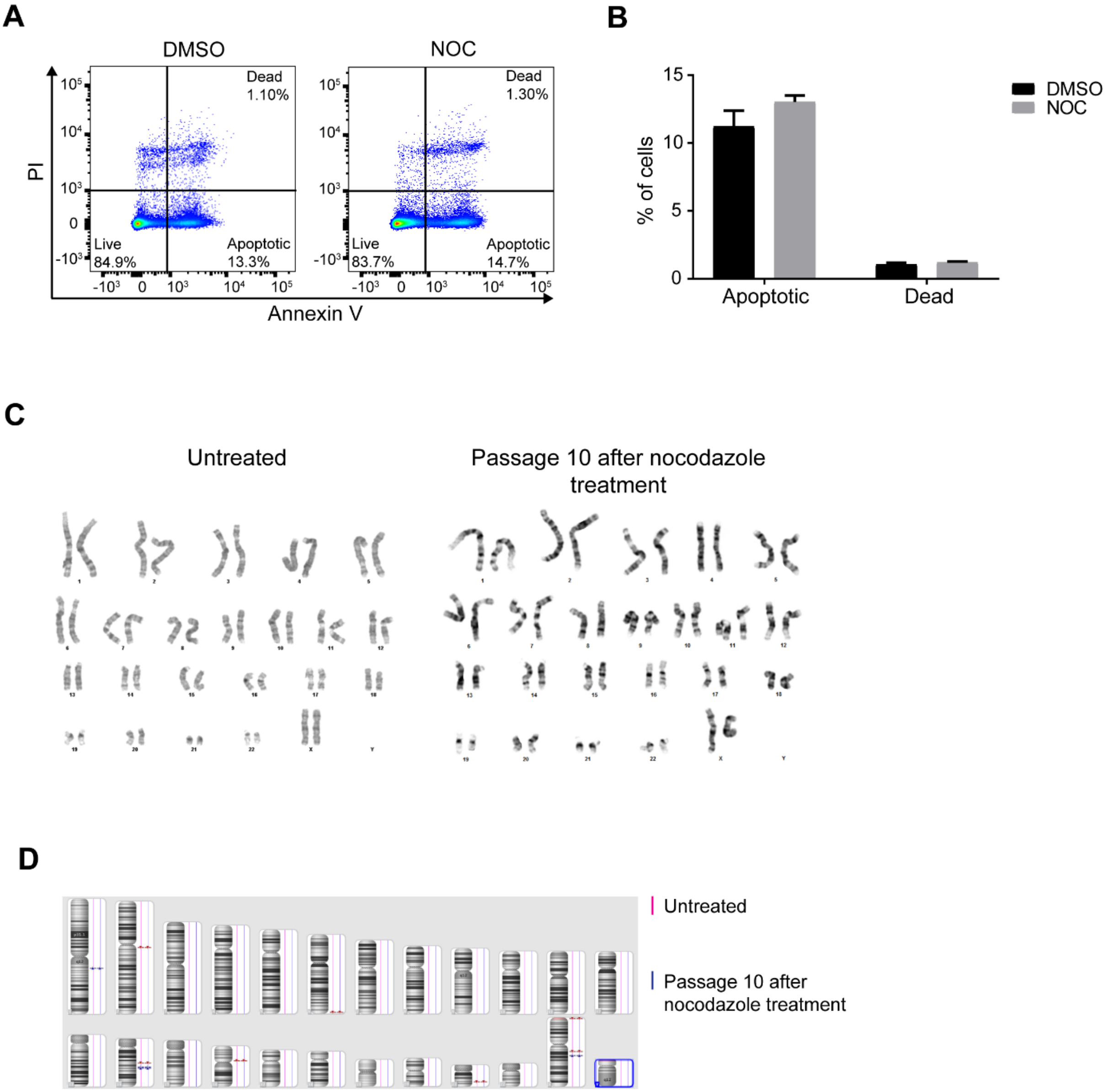

Figure S2. Nocodazole treatment does not cause karyotypic abnormalities in hESCs. Related to Figure 3.
(A) Representative flow cytometry analysis for Annexin V and Propidium iodide (PI) positive cells. Annexin V+/PI- cells are apoptotic and Annexin V+/PI+ cells are dead.
(B) Bar graph summarising flow cytometry results of Annexin V/PI staining. Error bars represent ±SEM of two independent experiments.
(C) Chromosomal spreads showing normal karyotype in untreated and nocodazole-treated H9 hESCs after ten passages in culture.
(D) Karyoview representation of CytoScan 750K array analysis comparing untreated (pink) and nocodazole treated (blue) H9 hESCs after ten passages in culture.

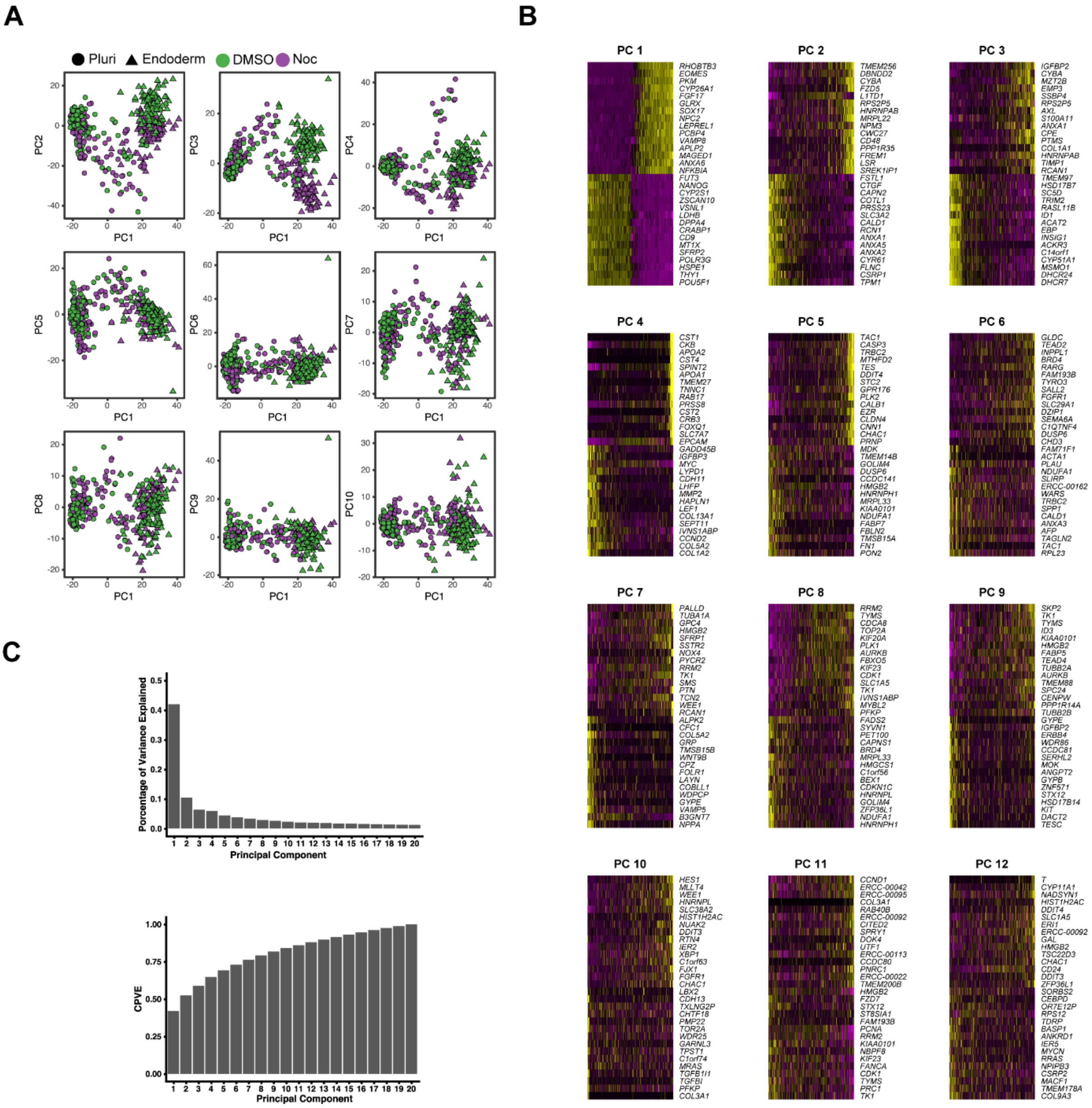

Figure S3. Single Cell RNA-Seq Confirms That Nocodazole Treatment Does Not Affect the Ability of Pluripotent Cells to Differentiate into Definitive Endoderm. Related to Figure 4.

(A) Principal component analysis (PCA) plot showing the assignment of cells based on differentiation and synchronization status. Normalized log-expression values were used. Dots represent individual cells. (DMSO = Green, Noc = Purple, Und = Circle, Endoderm = Triangle).

(B) Heatmaps showing the top 500 cells and 30 genes sorted by their first 12 principal component scores. These plots allow for visualisation of sources of heterogeneity in the dataset.

(C) Graphs showing the percentage of variance explained and the cumulative proportion of variance explained (CPVE).

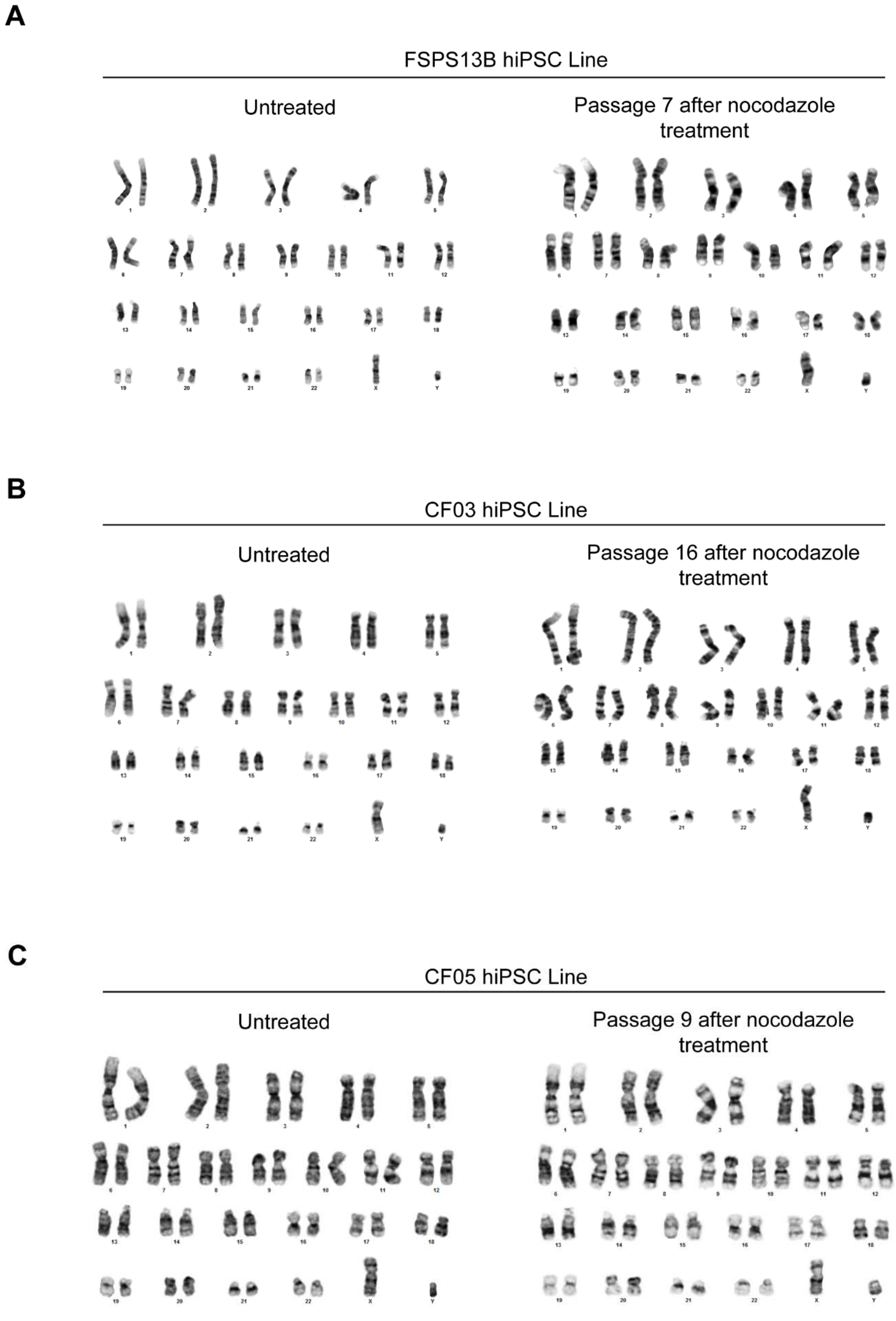

Figure S4. Nocodazole treatment does not cause karyotypic abnormalities in hiPSCs. Related to Figure 7. (A-C) Chromosomal spreads showing normal karyotype for both untreated and nocodazole treated FSPS13B line (A), CF03 line (B), and CF05 line (C).

Supplemental Experimental Procedures

Germ layer Differentiation
Mesoderm subtypes were generated in a 2-step protocol as previously described (Mendjan et al., 2014). For lateral plate mesoderm (LPM) formation, cells were cultured for 36 hours in CDM-PVA supplemented with 20ng/ml FGF2, 10µM LY294002 (Promega) and 10ng/ml BMP4 (R&D). Subsequently cells were cultured for 3 days in CDM-PVA supplemented with 20ng/ml FGF2 and 50ng/ml BMP4 changing medium every two days. For cardiac mesoderm (CM) formation, cells were cultured for 36 hours in CDM-BSA (without insulin) supplemented with 20ng/ml FGF2, 10µM LY294002, 10ng/ml BMP4 and 50ng/ml Activin A (Dr. Marko Hyvönen, Cambridge University). Subsequently cells were cultured for 4 days in CDM-BSA (without insulin) supplemented with 8ng/ml FGF2, 10ng/ml BMP4, 1µM IWR1 (WNT signalling inhibitor; Tocris Bioscience) and 0.5µM Retinoic Acid (Sigma-Aldrich), changing medium every two days. For presomitic mesoderm (PSM) formation, cells were cultured for 36 hours in CDM-BSA (without insulin) supplemented with 20ng/ml FGF2 and 8µM CHIR99021 (WNT signalling activator; Tocris Bioscience). Subsequently cells were cultured for 4 days in CDM-BSA (with insulin) supplemented with 4ng/ml FGF2, 1µM Retinoic Acid, 0.1µM LDN193189 (BMP signalling inhibitor; Sigma-Aldrich) and 10µM SB431542 (TGF-β signalling inhibitor; Tocris Bioscience). For mesoderm differentiation cells were plated on gelatin and mouse embryonic fibroblast (MEF) medium coated plates.

Definitive endoderm was generated using a 3-day protocol. On day 1 cells were cultured in CDM-PVA supplemented with 80ng/ml FGF2, 10µM LY294002, 10ng/ml BMP4, 100ng/ml Activin A and 3µM CHIR99021. On day 2 the cells were cultured in CDM-PVA supplemented with 80ng/ml FGF2, 10µM LY294002, 10ng/ml BMP4 and 100ng/ml Activin A. On day 3 the cells were cultured in RPMI-B27 media supplemented with 80ng/ml FGF2 and 100ng/ml Activin. For endoderm differentiation cells were plated on vitronectin coated plates (10µg/ml, Stem Cell Technologies).

Neuroectoderm was generated using a 7-day protocol. On days 1 and 2 the cells were cultured in CDM-BSA supplemented with 20ng/ml FGF2, 3µM CHIR99021, 0.1µM LDN193189 and 10µM SB431542, changing medium daily. From day 3 to day 6 cells were cultured in Neurobasal medium/DMEM F12 (1:1 ratio) supplemented with N2-B27, 1% glutamine, 10µM SB431542 and 0.7% β-mercaptoethanol, changing medium daily.

Smooth Muscle Cell Differentiation
For smooth muscle cell formation, LPM cells were dissociated with TrypLE Express (Life Technologies) for 5 minutes at 37ºC, washed once with CDM-PVA and centrifuged at 200g for 3 minutes. Cells were seeded on gelatin and MEF medium coated plates at a density of $2.6x10^4$ cells/cm^2 in CDM-PVA supplemented with 10ng/ml PDGF-BB (Peprotech) and 2ng/ml TGF-β (Peprotech) for 12 days. Media was changed every two days and cells split when confluent at a 1:2 ratio, usually on day 3 or day 6 (Cheung et al., 2012).

Cardiomyocyte Differentiation
Following cardiac mesoderm formation, cells were cultured for two days in CDM-BSA (with insulin) supplemented with 8ng/ml FGF2 and 10ng/ml BMP4 (R&D) and subsequently fed every two days with CDM-BSA (with insulin). Onset of beating was observed on day 7-9 of differentiation (Mendjan et al., 2014).

Chondrocyte Differentiation
Following presomitic mesoderm formation, cells were cultured in CDM-BSA (with insulin) supplemented with 8ng/ml FGF2 and 10ng/ml BMP4 (R&D) for 10 days, changing media every two days (Mendjan et al., 2014).

Hepatocyte Differentiation
Protocol for hepatocyte formation has been modified from (Gieseck et al., 2015). Following definitive endoderm formation, cells were cultured for 5 days in RPMI-B27 supplemented with 50ng/ml Activin-A to generate foregut, changing medium daily. Subsequently cells were cultured for 13 days in Hepatozyme supplemented with 20ng/ml OSM (R&D) and 50ng/ml HGF (Peprotech) to generate mature hepatocytes, changing media every two days.

Small Molecule Cell Cycle Inhibitor Treatment
H9 hESCs were treated with the small molecule inhibitors and a number of different doses were tested. The inhibitors used are as follows:

Small Molecule Cell Cycle Inhibitors Used

Inhibitor	Concentrations used	Catalogue Number	Supplier
Aphidicolin	15ng/ml, 75ng/ml	A4487	Sigma-Aldrich
Colcemid	2μg/ml	15212012	Invitrogen
Hydroxyurea	20μM, 100μM, 200μM	H8627	Sigma-Aldrich
L-Mimosine	5μM, 10μM, 50μM	M0253	Sigma-Aldrich
Lovastatin	4μM, 8μM, 16μM	438185	Millipore
Nocodazole	0.02μg/ml, 0.1μg/ml, 0.2μg/ml	M1404	Sigma-Aldrich
Thymidine	400μM, 800μM	T9250	Sigma-Aldrich

Cells were treated with the inhibitors for 16 or 24 hours. For inhibitor removal, cells were washed twice with E8 media and fed normally with maintenance media in the absence of the inhibitors.

Flow Cytometry

For flow cytometry analysis, cells were dissociated into a single cell suspension by incubating with cell dissociation buffer (CDB; Gibco) for 10 minutes at 37ºC and washed once with 1% BSA-PBS. Cells were fixed and permeabilised using the BD Cytofix/Cytoperm solution for 20 minutes at 4ºC (BD Biosciences). After one wash with the perm/wash buffer (BD Biosciences), cells were blocked in perm/wash buffer with 10% donkey serum (Bio-Rad) and 0.1% Triton X-100 (Sigma-Aldrich) for 30 minutes at room temperature. Cells were then stained with the mouse OCT3/4 antibody (C-10; sc5279; Santa Cruz) at 1:200 dilution in perm/wash buffer with 0.1% Triton X-100 for 1 hour at room temperature. Following two washes with the same buffer, cells were incubated with the secondary antibody Alexa Fluor® 647 goat anti-mouse IgM (1:1,000, A21236; Invitrogen) for 1 hour at room temperature in the same buffer, protected from light. Cells were analysed on a Cyan ADP flow cytometer and FlowJo software.

Apoptosis Analysis

Apoptosis was assessed using the Annexin V/Dead Cell Apoptosis kit (Invitrogen) according to the manufacturer's instructions. Briefly, cells were gently dissociated with accutase (Gibco) for 5 min, and washed in E8 media followed by one wash with 1% BSA in PBS. Cells were resuspended in 100 µl 1x binding buffer and mixed with 5 µl of FITC Annexin V and 1 µl propidium iodide for 15 minutes at room temperature. After addition of 400 µl of 1× binding buffer, cells were analysed on a FACS Canto II (BD Biosciences) flow cytometer and FlowJo software.

Western Blot

For protein isolation, cells were washed once with PBS and harvested with cell dissociation buffer (CDB; Gibco) for 10 minutes at 37°C and washed once with cold 1% BSA-PBS. Pellets were collected by centrifugation at 4ºC and 300g for 3 minutes and lysed with RIPA buffer supplemented with protease and phosphatase inhibitors (Roche) for 30 minutes on ice, vortexing every 10 minutes. Following lysis, samples were centrifuged at 4ºC and 17,000g for 5 minutes and the supernatant collected. Protein was quantified using the Pierce BCA Protein Assay Kit (Thermo Fisher Scientific) according to the manufacturer's instructions. Samples were prepared for Western blot analysis by adding 1x NuPAGE LDS Sample Buffer (Thermo Fisher Scientific) and 1% β-mercaptoethanol and boiling the samples at 95ºC for 5 minutes. 10-35 µg of protein was loaded in 4-12% NuPAGE Bis-Tris Precast Gels (Thermo Fisher Scientific) and run using NuPAGE MOPS SDS Running Buffer (Thermo Fisher Scientific). For identification of the size of the target protein, Precision Plus Protein Ladder was used (Bio-Rad). Protein was transferred on PVDF membranes (Bio-Rad) by liquid transfer using NuPAGE Transfer Buffer (Thermo Fisher Scientific). Membranes were blocked using 4% non-fat dried milk in Tris-buffered saline and 0.1% Tween buffer (TBST buffer) for 30 minutes and incubated with primary antibody overnight at 4ºC in TBST. The primary antibodies used are: Cyclin D1 (2922S, Cell Signalling Technology, 1: 1,000), Cyclin D2 (3741S, Cell Signalling Technology, 1: 1,000), Cyclin D3 (2936S, Cell Signalling Technology, 1: 1,000) and α-tubulin (T9026, Sigma-Aldrich, 1: 40,000). Following 3 washes with TBST, membranes were incubated with horseradish peroxidase (HRP)-conjugated secondary antibody for 1 hour at room temperature. Membranes were then washed 3 times with TBST and incubated with Pierce ECL Western Blotting Substrate and exposed to X-Ray Super RX Films (Fujifilm).

RNA Extraction, cDNA Synthesis and qRT-PCR

Total RNA was extracted using the GenElute™ Mammalian Total RNA Miniprep Kit (Sigma-Aldrich) and the On-Column DNase I Digestion set (Sigma-Aldrich) according to the manufacturer's instructions. RNA was reverse transcribed using 250ng random primers (Promega), 0.5mM dNTPs (Promega), 20U RNAseOUT, 0.01M DTT and 25U of SuperScript II (all from Invitrogen). The resulting cDNA was diluted 30-fold for the qPCR

reaction. Quantitative PCR mixtures were prepared using the KAPA SYBR® FAST qPCR Master Mix (2X) Kit (Kapa Biosystems), 4.2µl of cDNA and 200nM of each of the forward and reverse primers. Samples were run on 384 well plates using the QuantStudio 12K Flex Real-Time PCR System machine and results analysed using the delta-delta cycle threshold method (ΔΔCt). Expression values were normalized to the housekeeping gene Porphobilinogen Deaminase (*PBGD*).

qPCR Primers Used

Gene	Forward Primer (5'-3')	Reverse Primer (5'-3')
ACAN	CCCCTGCTATTTCATCGACCC	GACACACGGCTCCACTTGAT
A1AT	CCACCGCCATCTTCTTCCTGCCTGA	GAGCTTCAGGGGTGCCTCCTCTG
ACTN1	CAAACCTGACCGGGGAAAAAT	CTGAATAGCAAAGCGAAGGATGA
ALB	CCTTTGGCACAATGAAGTGGGTAACC	CAGCAGTCAGCCATTTCACCATAG
CNN1	GTCCACCCTCCTGGCTTT	AAACTTGTTGGTGCCCATCT
COL2A1	TGGACGCCATGAAGGTTTTCT	TGGGAGCCAGATTGTCATCTC
EOMES	ATCATTACGAAACAGGGCAGGC	CGGGGTTGGTATTTGTGTAAGG
NANOG	CATGAGTGTGGATCCAGCTTG	CCTGAATAAGCAGATCCATGG
PAX6	CTTTGCTTGGGAAATCCGAG	AGCCAGGTTGCGAAGAACTC
PBGD	GGAGCCATGTCTGGTAACGG	CCACGCGAATCACTCTCATCT
POU5F1 (OCT4)	AGTGAGAGGCAACCTGGAGA	ACACTCGGACCACATCCTTC
SOX1	Quantitect primers (Qiagen)	Quantitect primers (Qiagen)
SOX17	CGCACGGAATTTGAACAGTA	GGATCAGGGACCTGTCACAC
SOX2	TGGACAGTTACGCGCACAT	CGAGTAGGACATGCTGTAGGT
BRACHYURY	TGCTTCCCTGAGACCCAGTT	GATCACTTCTTTCCTTTGCATCAAG
TAGLN	TCTTTGAAGGCAAAGACATGG	TTATGCTCCTGCGCTTTCTT
TNNT2	ACAGAGCGGAAAAGTGGGAAG	TCGTTGATCCTGTTTCGGAGA

Immunostaining

For immunostaining analysis, cells were fixed for 20 minutes at 4ºC with 4% paraformaldehyde (PFA) in PBS and washed once with PBS. Cells were subsequently blocked and permeabilised at room temperature for 30 minutes in PBS with 4% donkey serum (Bio-Rad) and 0.1% Triton X-100 (Sigma-Aldrich). Primary antibodies were diluted in the same buffer and incubate with the cells for 2 hours at room temperature or overnight at 4ºC. After three washes with PBS, cells were incubated with AlexaFluor secondary antibodies for 1 hour at room temperature protected from light. Cells were subsequently washed three times for 5 minutes with PBS, adding Hoechst 33258 (bis-Benzimide H, 1: 10,000 dilution; Sigma-Aldrich) during the first wash to stain nuclei.

Antibodies Used for Immunostaining Analysis

Antibody	Species	Dilution	Catalogue Number	Manufacturer
BRACHYURY	Goat	1:200	AF2085	R&D Systems
EOMES	Rabbit	1:500	ab183991	Abcam
HAND1	Goat	1:200	AF3168	R&D Systems
NANOG	Goat	1:200	AF1997	R&D Systems
OCT3/4 (C-10)	Mouse	1:200	sc-5279	Santa Cruz Biotechnology
SOX1	Goat	1:200	AF3369	R&D Systems
SOX17	Goat	1:200	AF1924	R&D Systems
SOX2	Goat	1:200	AF2018	R&D Systems

Alexa Fluor 488 donkey anti-goat	-	1:1,000	A11055	Invitrogen
Alexa Fluor 488 donkey anti-mouse	-	1:1,000	A21202	Invitrogen
Alexa Fluor 488 donkey anti-rabbit	-	1:1,000	A21206	Invitrogen
Alexa Fluor 647 donkey anti-goat	-	1:1,000	A21447	Invitrogen
Alexa Fluor 647 donkey anti-mouse	-	1:1,000	A31571	Invitrogen
Alexa Fluor 647 donkey anti-rabbit	-	1:1,000	A31573	Invitrogen

Alcian Blue Staining of Chondrocytes

Monolayer cultures of chondrocytes were fixed with 4% paraformaldehyde (PFA) for 20 minutes at 4ºC. Cells were then washed with 0.5N HCl and stained overnight with 0.25% (w/v) Alcian Blue 8GX (Sigma-Aldrich) in 0.5N HCl. Stained cells were visualized using a Leica dissecting microscope. Alcian Blue dye was solubilized by overnight incubation with 8M guanidine hydrochloride (Sigma-Aldrich) and quantified by absorbance at 595nm using a spectrophotometer.

SMC Contraction Assay

SMC contraction was induced by treatment with 100µM carbachol (Sigma-Aldrich). Images were acquired with EVOS FL imaging system (Thermo Fisher Scientific) prior to the addition and ten minutes after the addition of carbachol. The surface area of the cells was measured using ImageJ software.

CYP3A4 Assay on Hepatocytes

CYP3A4 activity of the hepatocytes was detected in hepatocytes using the P450-Glo CYP3A4 Assay (Promega) according to the manufacturer's instructions. Luminescence was measured using a GloMax 96 Microplate Luminometer (Promega).

Karyotype and CytoScan Analyses

Cells were treated with 0.1µg/ml colcemid (Gibco) supplemented with 10µM Y27632 (ROCK inhibitor) for 4 hours. Cells were then harvested with cell dissociation buffer (CDB; Gibco) for 10 minutes at 37ºC and centrifuged at 1200rpm for 7 minutes. 0.055M of KCl hypotonic solution was added to the cells and incubated at 37ºC for 30 minutes to allow the cells to swell and release the chromosomes. Following centrifugation at 1200rpm for 7 minutes, cells were fixed with 2ml methanol:glacial acetic acid at a 3:1 ratio. Fixed cell suspensions were sent to the Cytogenetics Laboratory, Cambridge University Hospitals, UK and karyotype analysis was performed by standard G banding techniques to confirm euploidy of the cell lines. For the in-depth CytoScan characterisation, genomic DNA was isolated using the GenElute Mammalian Genomic DNA Miniprep Kit (Sigma-Aldrich). 1µg of genomic DNA was sent to the Cytogenetics Laboratory, Cambridge University Hospitals, UK and analysis was performed using CytoScan 750K Array Kit.

Supplemental References

Cheung, C., Bernardo, A.S., Trotter, M.W.B., Pedersen, R.A., and Sinha, S. (2012). Generation of human vascular smooth muscle subtypes provides insight into embryological origin–dependent disease susceptibility. Nat. Biotechnol. *30*, 165–173.

Gieseck, R.L., Vallier, L., and Hannan, N.R.F. (2015). Generation of Hepatocytes from Pluripotent Stem Cells for Drug Screening and Developmental Modeling. In Protocols in In Vitro Hepatocyte Research, M. Vinken, and V. Rogiers, eds. (New York, NY: Springer New York), pp. 123–142.

Mendjan, S., Mascetti, V.L., Ortmann, D., Ortiz, M., Karjosukarso, D.W., Ng, Y., Moreau, T., and Pedersen, R.A. (2014). NANOG and CDX2 pattern distinct subtypes of human mesoderm during exit from pluripotency. Cell Stem Cell *15*, 310–325.
